# JUNO-coated beads as a functional assay to capture and characterize fertilization-competent human sperm

**DOI:** 10.1093/hropen/hoag010

**Published:** 2026-02-13

**Authors:** Paula Cots-Rodríguez, Xinyin Wang, Mirian Sanchez-Tudela, Karen K Siu, Patrick Yip, Emilio Gómez, Jeffrey E Lee, Julieta G Hamze, Maria Jiménez-Movilla

**Affiliations:** Department of Cell Biology and Histology, Faculty of Medicine, CEIR Campus Mare Nostrum (CMN), University of Murcia, Murcia, Spain; Instituto Murciano de Investigación Biosanitaria Pascual Parrilla (IMIB-Arrixaca), Murcia, Spain; Department of Laboratory Medicine and Pathobiology, Temerty Faculty of Medicine, University of Toronto, Toronto, ON, Canada; Department of Cell Biology and Histology, Faculty of Medicine, CEIR Campus Mare Nostrum (CMN), University of Murcia, Murcia, Spain; Department of Laboratory Medicine and Pathobiology, Temerty Faculty of Medicine, University of Toronto, Toronto, ON, Canada; Department of Laboratory Medicine and Pathobiology, Temerty Faculty of Medicine, University of Toronto, Toronto, ON, Canada; Department of Cell Biology and Histology, Faculty of Medicine, CEIR Campus Mare Nostrum (CMN), University of Murcia, Murcia, Spain; IVF Laboratory, Next Fertility/Gametia, Murcia, Spain; Department of Laboratory Medicine and Pathobiology, Temerty Faculty of Medicine, University of Toronto, Toronto, ON, Canada; Department of Cell Biology and Histology, Faculty of Medicine, CEIR Campus Mare Nostrum (CMN), University of Murcia, Murcia, Spain; Instituto Murciano de Investigación Biosanitaria Pascual Parrilla (IMIB-Arrixaca), Murcia, Spain; Department of Cell Biology and Histology, Faculty of Medicine, CEIR Campus Mare Nostrum (CMN), University of Murcia, Murcia, Spain; Instituto Murciano de Investigación Biosanitaria Pascual Parrilla (IMIB-Arrixaca), Murcia, Spain

**Keywords:** fertilization, spermatozoa selection, spermatozoa vitrification, *in vitro* sperm-binding model, human-based model, fertilization-competent spermatozoa, acrosome reaction, DNA integrity, JUNOIZUMO1

## Abstract

**STUDY QUESTION:**

Can human fertilization-competent spermatozoa be captured through their ability to bind the oocyte receptor JUNO?

**SUMMARY ANSWER:**

JUNO-coated beads, which mimic the oocyte geometry, selectively bound acrosome-reacted spermatozoa with intact DNA, revealing that vitrification preserves functional sperm binding while slow cryopreservation increases non-specific interactions.

**WHAT IS KNOWN ALREADY:**

It is well established that sperm must undergo the acrosome reaction and expose the receptor IZUMO1 on the sperm head to bind specifically to JUNO on the oolemma. Studying the spermatozoa that reaches and engages with the oolemma, however, remains highly challenging due to the technical difficulty of recovering these sperm at the site of the molecular interaction. Bead-based models that contain oocyte receptors have therefore emerged as a powerful approach to functionally assess sperm–oocyte interactions, with promising applications for evaluating sperm quality.

**STUDY DESIGN, SIZE, DURATION:**

This was a cross-sectional experimental study including 21 semen donors of reproductive age recruited between January 2023 and June 2025. The JUNO-bead-based model was first validated using fresh human semen samples to establish the optimal sperm concentration and co-incubation time. Subsequently, two semen preservation methods, slow freezing and rapid freezing, were compared with respect to sperm-binding capacity to JUNO-coated beads, acrosomal status and DNA integrity. Finally, donors were classified according to sperm-binding capacity and validated by the hamster test.

**PARTICIPANTS/MATERIALS, SETTING, METHODS:**

Recombinant JUNO protein was secreted and purified from *Drosophila melanogaster* S2 cultures. Digital SPR was used to confirm JUNO–IZUMO1 binding kinetics and imaging flow cytometry was performed to assess the biological activity of recombinant JUNO. Protein–bead conjugation and activity were verified by immunochemistry and western blot. Human semen samples were obtained from donors aged 19–42 years, including both fresh ejaculates and cryopreserved samples. Sperm-binding capacity, acrosome reaction, and DNA fragmentation were analysed using widefield fluorescence microscopy and flow cytometry, and the specificity of sperm–bead interaction was evaluated with anti-IZUMO1 monoclonal antibodies.

**MAIN RESULTS AND THE ROLE OF CHANCE:**

Recombinant JUNO bind human IZUMO1 ectodomain with a KD = 38 nM and specifically recognizes acrosome-reacted sperm and not acrosome-intact sperm. Human JUNO recombinant protein was successfully conjugated to oocyte-sized beads to generate a sperm-binding assay mimicking the geometry of the oocyte and experimental conditions of IVF. Human sperm bound specifically to JUNO-beads in a dose- and time-dependent manner, with highly significant differences compared to control beads (*P *≤ 0.0001). Vitrified-based cryopreserved sperm displayed higher binding to JUNO-beads than conventionally cryopreserved samples (*P *≤ 0.0001). Binding was significantly inhibited by an anti-IZUMO1 (2.5 µg/mL) antibody that blocks specifically the IZUMO1–JUNO interaction in vitrified samples (*P* ≤ 0.01), but not in conventionally cryopreserved sperm. Sperm bound to JUNO-beads were predominantly acrosome-reacted in both preservation methods; however, vitrified samples retained higher DNA integrity compared with conventionally cryopreserved samples. The assay proved robust across multiple donors and ejaculates, allowing classification into low- and high-binding capacity (LBC and HBC) groups, and data were validated using the hamster test. Pearson correlation analyses revealed only weak associations between total sperm motility and bead-binding parameters (|r| < 0.27), indicating negligible or absent linear relationships.

**LARGE-SCALE DATA:**

N/A

**LIMITATIONS, REASONS FOR CAUTION:**

This study was performed *in vitro*, and the number of semen donors was limited. As all participants were healthy donors, the population represents a selected fertile subpopulation. Further studies using samples from diverse patient populations are required to validate the assay’s potential as a predictor of male fertility. While sperm–egg binding is an essential prerequisite for fertilization, the JUNO-bead–based assay focuses on this initial interaction and does not capture downstream fertilization events.

**WIDER IMPLICATIONS OF THE FINDINGS:**

This study positions the JUNO-bead binding assay as a powerful functional model to investigate the biology of fertilization-competent sperm. By selectively capturing spermatozoa that have undergone the acrosome reaction and maintain DNA integrity, the model provides a unique experimental platform to study the molecular determinants of fertilization, to refine the selection of sperm for assisted reproduction, and to identify potential targets for novel contraceptive strategies. Beyond preservation protocols, these findings provide new functional evidence that sperm preservation method directly influences the molecular integrity required for fertilization, supporting vitrification as a superior approach over slow freezing. Moreover, the JUNO-bead assay emerges as a sensitive tool to reveal differences in sperm quality that are not captured by standard semen analysis, with potential applications in the optimization of assisted reproduction and fundamental research on the mechanisms that define the fertilizing spermatozoon.

**STUDY FUNDING/COMPETING INTEREST(S):**

This work is part of the projects PID 2020-114109GB-I00 and PID2024-159920OB-I00 funded by MICIU/AEI/10.13039/501100011033 and by ERDF, EU and 23046/GERM/25 funded by FSRM/10.13039/100007801 to M.J.-M. This work was also supported, in part, by the Gates Foundation [INV-055841]. The conclusions and opinions expressed in this work are those of the author(s) alone and shall not be attributed to the Foundation. Under the grant conditions of the Foundation, a Creative Commons Attribution 4.0 License has already been assigned to the Author Accepted Manuscript version that might arise from this submission. Protein production and characterization and biophysics infrastructure is supported by funding from a Canadian Institutes of Health Research Project Grant (PJT-203841) and Canada Foundation for Innovation John R Evans Leaders Fund (CFI-JELF) to J.E.L. The authors declare no conflicts of interest.

WHAT DOES THIS MEAN FOR PATIENTS? Identifying and studying the sperm cell that is truly capable of fertilizing an oocyte (egg) is a major challenge, because this process occurs precisely when the sperm binds to the oocyte and penetrates it. In this study, we developed a model based on microscopic beads that mimic the shape of the oocyte and are coated with JUNO, a receptor essential for sperm–oocyte recognition. These JUNO-beads capture only those sperm cells with the right molecular and cellular properties to bind to the oocyte membrane and trigger the first events leading to fertilization. Using this system, we compared sperm samples preserved through slow freezing (cryopreservation) or rapid freezing (vitrification). We observed that vitrified samples retained a higher proportion of sperm with fertilizing characteristics, that is, sperm that had undergone the acrosome reaction but maintained intact DNA. Furthermore, by applying this model to samples from different donors, we were able to classify them according to their high- or low-binding capacity to JUNO-beads.

Overall, this approach provides a new way to ‘capture’ and evaluate fertilizing sperm, offering potential applications for improving sperm quality assessment in assisted reproduction and a valuable tool for studying the defining features of the fertilizing sperm cell.

## Introduction

The fertilization process relies on a well-coordinated recognition mechanism between two highly specialized haploid cells: the spermatozoon and the oocyte (Okabe, 2013). Over the past years, several molecular factors involved in gamete interaction and fusion have been identified. On the sperm side, IZUMO1 plays a key role in the fusion process, as demonstrated by IZUMO1 knockout model, where *Izumo1*^−/−^ spermatozoa, despite their ability to penetrate the zona pellucida (ZP), fail to bind and fuse with the oolemma (Inoue *et al.*, 2005; Matsumura *et al.*, 2021). A similar phenotype has been described for DCST1, DCST2, FIMP, SOF1, SPACA6, TMEM81, and TMEM95 proteins, where each knockout model exhibited spermatozoa accumulating in the perivitelline space with impaired fusion ability with oolemma (Lorenzetti *et al.*, 2014; Barbaux *et al.*, 2020; Fujihara *et al.*, 2020; Lamas-Toranzo *et al.*, 2020; Noda *et al.*, 2020; Inoue *et al.*, 2021; Deneke *et al.*, 2024).

Conversely, only two factors have been identified on the oocyte side; CD9 and JUNO. Notably, the interaction between sperm IZUMO1 and oocyte JUNO represents the only well-documented interaction between human gametes (Bianchi *et al.*, 2014; Aydin *et al.*, 2016; Kato *et al.*, 2016; Ohto *et al.*, 2016; Jean *et al.*, 2019). The crucial role of JUNO in fertilization was demonstrated using *Juno*^−/−^ oocytes, which allowed penetration of the ZP by wild-type sperm but failed to support binding or fusion with the oolemma (Bianchi *et al.*, 2014). Similarly, in experiments using human zona-free oocytes, the presence of an anti-hJUNO antibody prevents human sperm from fusing with and fertilizing the oocyte (Jean *et al.*, 2019).

In this context, from ejaculation to fertilization, spermatozoa undergo physiological transformations, including capacitation, acrosome reaction, and crucial relocation of the IZUMO1 protein, all essential for successful interaction with JUNO and thus, fertilization (Yanagimachi, 1994; Inoue *et al.*, 2005; Puga Molina *et al.*, 2018). Nevertheless, the identification and analysis of fertilization-competent spermatozoa remain technically challenging. Current human sperm characterization methods primarily assess viability, morphology, and motility (World Health Organization, 2021). However, a significant proportion of male infertility cases remain idiopathic or unexplained (Ventimiglia *et al.*, 2021; Corsini *et al.*, 2023), underscoring the need for novel techniques to evaluate the fertilization capacity of apparently normal spermatozoa based on their molecular ability to bind the oocyte.

As a complementary approach to assisted reproductive technologies, sperm cryopreservation represents a crucial strategy for preserving male fertility. To ensure the effectiveness of this technique, it is essential to maintain both sperm viability and functionality after thawing (World Health Organization, 2021). Currently, two main sperm freezing methods exist: slow freezing and rapid freezing. Hereafter, slow freezing will be referred to as cryopreservation, and rapid freezing as vitrification. These methods differ in cooling rates, cryoprotectant composition, sample volume, storage devices, and thawing protocols (Li *et al.*, 2019; Tao *et al.*, 2020). Although vitrification has shown promising results, further evaluation of its impact on sperm fertilization capacity is required.

One of the major challenges in assessing the fertilization potential of both fresh and preserved sperm is the limited availability of human oocytes for IVF assays. For this reason, alternative methodologies have been developed to evaluate the sperm–oocyte binding capacity. The human ZP binding assay tests sperm fertilizing potential by measuring their ability to bind to isolated human zonae pellucidae, showing a correlation with IVF outcomes (Burkman *et al.*, 1988; Liu *et al*., 2003); however, limitations in sample accessibility persist. The zona-free hamster oocyte penetration test evaluates sperm function by testing their ability to undergo capacitation and the acrosome reaction, and to fuse with the oolemma (Yanagimachi *et al.*, 1976). However, due to its lack of physiological relevance, it may yield false negatives, as some sperm that fail this assay can still fertilize human oocytes *in vitro* and *in vivo* (‘Group Discussion’, 1986).

Given these limitations, both tests have been classified as obsolete in the latest edition of WHO Laboratory Manual for the Examination of Human Semen (World Health Organization, 2021). To address these shortcomings, a three-dimensional (3D) *in vitro* model of sperm–oocyte interaction has been developed (Hamze *et al.*, 2020a,b,c). This system uses magnetic Sepharose microbeads (hereafter referred to as ‘beads’) coated with recombinant oocyte proteins, such as ZP proteins (Hamze *et al.*, 2020b) and JUNO (Hamze *et al.*, 2020c), to mimic the size, shape, and molecular surface characteristics of the oocyte. The model has been previously validated in non-human species, including bovine and porcine. In the present study, we optimized a bead-based sperm-binding assay specifically for human sperm by employing microbeads coated with recombinant human JUNO. This approach allows for the evaluation of sperm-binding capacity to JUNO without the need for live human oocytes. Once validated, the model was applied to compare the fertilizing potential of spermatozoa preserved using cryopreservation versus vitrification techniques.

## Materials and methods

### Ethical approval

This study was approved and supervised by the Research Ethical committee (CEI 3094/2020) and Experimental Biosecurity committee (CBE 371/2020 and CBE 576/2023) of Universidad de Murcia. The human sperm imaging flow cytometry assay was approved by the University of Toronto Research Ethics Board (RIS protocol #44517) and TAHSN Sinai Health Research Ethics Board (protocol 22-0164-E). All participants provided written informed consent prior to inclusion in the study.

### Recombinant protein design and expression

The expression and purification of recombinant human JUNO and IZUMO1 ectodomains were performed following the protocol previously described in Aydin *et al.* (2016). Codon-optimized DNA sequences encoding JUNO (Uniprot: A6ND01, residues 20–228) and IZUMO1 (Uniprot: Q8IYV9, residues 22–254), each with a N-terminal BiP signal peptide and C-terminal thrombin cleavage site plus 10×-His affinity tag, were codon optimized for expression in *Drosophila melanogaster*, gene synthesized and subcloned into a modified pMT-puromycin expression vector (Invitrogen, Carlsbad, CA, USA). Plasmids were stably transfected in *Drosophila* S2 cells (Invitrogen) using Effectene transfection reagent (Qiagen, Venlo, Netherlands), according to the manufacturer’s protocol. Cells were adapted to serum-free Insect-XPRESS medium (Lonza Bioscience, Walkersville, MD, USA) with 6 μg/mL puromycin (BioShop Canada Inc, Burlington, ON, Canada) and expanded to 1 × 10^7^ cells/mL using vented 2 L shaker flasks (VWR, Radnor, PA, USA) at 27 °C, 120 rpm. Protein expression was induced with a final concentration of 500 μM sterile-filtered CuSO_4_ (Millipore Sigma, Burlington, MA, USA), and culture supernatants were harvested 6 days post-induction, clarified by centrifugation (6750 ×g for 20 minutes), concentrated and buffer exchanged into Ni-NTA binding buffer (20 mM Tris-HCl [pH 8.0], 300 mM NaCl, 20 mM imidazole) using a Centramate tangential flow filtration system (Pall Corp., Port Washington, NY, USA). His-tagged JUNO_20–228_ and IZUMO_122–254_ were purified by Ni-NTA affinity chromatography (Qiagen) followed by size exclusion chromatography (SEC) on a Superdex-200 Increase 10/300 column (Cytiva, Marlborough MA, USA) equilibrated with PBS or HBS (HEPES-buffered saline). For tag removal, Ni-NTA eluates were digested with thrombin (EMD Millipore, Burlington, MA, USA) at 22 °C for 24 hours (1U per mg of protein) during dialysis in 1× PBS, then subjected to SEC. Peak fractions were pooled and protein concentrations were quantified by measuring their absorbance at 280 nm.

### Surface plasmon resonance analysis of JUNO–IZUMO1 binding

Human JUNO ectodomain (2 mg/mL) was biotinylated using the EZ-link Sulfo-NHS-LC-Biotin kit (Thermo Scientific, Waltham, MA, USA) at RT for 45 minutes at a 1:1 molar ratio, followed by overnight dialysis into PBS (pH 7.4) at 4 °C. Single-cycle kinetic measurements were performed at 25 °C on the Nicoya Alto digital SPR system using a 16-channel carboxyl PEG cartridge (Nicoya, Kitchener, ON, Canada). The carboxy PEG cartridge was first coated with streptavidin and then biotinylated JUNO (800 nM) was immobilized as the bait. IZUMO1 (residues 22–254) was loaded as the analyte in a serial dilution series (1000 nM, 333 nM, 111 nM, 37 nM and 12.3 nM). The binding experiment was conducted in PBS (pH 7.4) containing 0.05% (v/v) Tween-20, with regeneration using 10 mM NaOH. Response maxima (R*_max_*) and χ^2^ were monitored for all runs. Data were processed using Nicosystem software (Alto version 2.5.2, Nicoya, Kitchener, ON, Canada). Experiments were performed in technical triplicate.

### Imaging flow cytometry analysis of human sperm bound JUNO

Human JUNO ectodomain (2 mg/mL) was buffer exchanged into 100 mM NaHCO_3_ (pH 8.3) and labelled with Alexa Fluor 647 NHS ester (Thermo Scientific) at a 1:2 protein: dye molar ratio for 1 hour at RT. Excess dye was removed by overnight dialysis into PBS (pH 7.4) at 4 °C.

Cryopreserved human donor semen (Fairfax Cryobank, Toronto, ON, Canada) was thawed, applied to an ORIGIO Gradient 40/80 (Cooper Surgical, Trumbull, CT, USA), and centrifuged at 500×g for 10 minutes. Purified sperm were capacitated at 1 × 10^6^/mL in ORIGIO Sperm Wash supplemented with 10 mg/ml human serum albumin for 3 hours at 37 °C, followed by induction of the acrosome reaction using a final concentration of 10 µM calcium ionophore for 15 minutes at 37 °C. Sperm were then stained with DAPI (Millipore Sigma), PNA-FITC (Millipore Sigma), and JUNO–Alexa Fluor 647 for 30 minutes in the dark. Fluorescence and brightfield images were acquired using an Amnis ImageStream Mark II imaging flow cytometer equipped with a high-gain camera at 60× magnification (Cytek Biosciences, Fremont, CA, USA). DAPI, FITC, and Alexa Fluor 647 were excited with 405 nm, 488 nm, and 592 nm lasers, respectively, and collected in separate imaging channels. A total of 50 000 events were measured. Data acquisition and analysis were performed using IDEAS 6.2 software (Cytek Biosciences).

### Conjugation of magnetic beads

The conjugation of recombinant protein to the magnetic beads was performed following the modified protocol previously described in Hamze *et al.* (2020a,c). Briefly, 40 µL of magnetic Sepharose beads (His Mag Sepharose Excel, GE Healthcare, Chicago, IL, USA) were size-selected (>70 µm) using an EASYstrainer™ 70 µm filter (Greiner Bio-One, Kremsmünster, Austria) and washed in an imidazole-containing buffer to ensure the absence of any bound proteins. The washed beads at a final concentration of 2 × 10^4^ beads/mL were then incubated overnight at 4 °C with either 3 µg of recombinant His-tagged JUNO protein (BJUNO), with untagged recombinant JUNO protein (BJUNOHis-off) and without protein (BControl) under orbital agitation. Following incubation, the conjugated beads were washed with sodium phosphate buffer and stored at 4 °C until use. SDS-PAGE was employed to confirm the successful conjugation of recombinant proteins to magnetic beads. Blots were incubated overnight at 4 °C with anti-His (1:1000 v/v; Qiagen) or rabbit anti-JUNO (1/5000 v/v; Biorbyt, Cambridge, UK) antibodies, and the membrane was incubated for 1 hour at RT with HRP-conjugated anti-mouse (1:10 000 v/v; Thermo Fisher Scientific) or anti-rabbit (10 000 v/v; Santa Cruz Biotechnology, Dallas, TX, USA) secondary antibodies, respectively. Immunoreactivity was revealed by chemiluminescence detection (Pierce ECL-Plus, Thermo Fisher Scientific). The details of all antibodies and kits used can be found in Supplementary Table S1.

To detect the IZUMO1 recombinant protein binding to BJUNO, purified rIZUMO1 (10 µg/mL) was added to BJUNO and BControl and incubated in G-IVF™ PLUS media (Vitrolife, Gothenburg, Sweden) for 1 hour (37 °C, 6% CO_2_). For immunochemistry, groups of 30 beads were fixed (2% paraformaldehyde for 30 minutes), blocked for 1 hour at RT (calcium- and magnesium-free PBS supplemented with 5% FCS), incubated overnight at 4 °C with rabbit anti-JUNO (1/100 v/v; Biorbyt) or anti-IZUMO1 4E04 (1/100 v/v; Tang *et al.*, 2022) antibodies and with the corresponding secondary antibody for 1 hour at RT (anti-rabbit IgG Alexa Fluor 555 (1:200 v/v; Invitrogen) or anti-mouse IgG Alexa Fluor 488 (1:200 v/v; Jackson ImmunoResearch, West Grove, PA, USA), respectively). Samples were then observed using widefield fluorescence microscopy (Widefield Leica Thunder-TIRF imager; Leica Microsystems, Wetzlar, Germany).

### Semen sample preparation

Only samples fulfilling the WHO reference values (World Health Organization, 2021) for sperm total motility provided either from the fertility clinic or the biobank were included in the study.

#### Fresh samples

Three fertile donors were recruited from Next Fertility Murcia clinic (Spain). Semen samples were obtained by masturbation following 3–5 days of sexual abstinence. After liquefaction at RT, the semen samples were washed in G-MOPS™ medium (1:1, v/v) and resuspended in a final volume of 2 mL of G-MOPS™ (Vitrolife). Semen parameters were determined following WHO guidelines (World Health Organization, 2021). Only samples that fulfilled the WHO reference values were included in the analysis (Supplementary Table S2).

#### Frozen samples

Two semen freezing methods, conventional cryopreservation and vitrification, were compared in this study. Semen samples from three different donors were used. Each ejaculate was processed by density gradient centrifugation, after which the resulting spermatozoa were divided and frozen using both techniques. The frozen samples were provided by BiokiBank (Vitoria-Gasteiz, Spain), which performed all cryopreservation and vitrification procedures.

For conventional cryopreservation, semen samples were mixed with the cryoprotectant SpermFreeze™ SSP (FERTIPRO, Beernem, Belgium) at a 1:3 (v/v) ratio, and then slowly frozen in straws following the standard laboratory protocol. For vitrification, semen samples were processed using the patented VitriStraw^®^ technology (WO 2012/028967, BiokiBank). Briefly, following density gradient centrifugation, the vitrification medium was added to the spermatozoa, and the samples were subjected to ultra-rapid cooling in liquid nitrogen.

Thawing of spermatozoa was performed according to the supplier’s recommendation. For vitrified samples, 4 mL of pre-heated (42 °C) G-MOPS™ PLUS medium (Vitrolife) was used, and the sample was left at 42 °C for 5 minutes before further processing. For the cryopreservation method, the straws were thawed in a water bath at 40 °C for 5 minutes, and the content was then transferred then into 4 mL of G-MOPS™ PLUS medium (Vitrolife).

Motile spermatozoa were selected from both fresh and frozen samples using the swim-up method for 1 hour at 37 °C in supplemented G-MOPS™ PLUS medium (20 mM CaCl_2_ and 10 µg/mL progesterone). Motility was assessed using a computer-assisted sperm analysis (CASA) system (ISAS^®^; Proiser, Valencia, Spain) at 37 °C. A minimum of five fields and 200 spermatozoa per sample were analysed (52 frames/s, 10× magnification objective and Spermtrack20 chamber). Selected spermatozoa were further capacitated for three additional hours under the same conditions before use.

### Sperm-binding assay and acrosome state assessment

Groups of 30–35 magnetic sepharose beads were washed in equilibrated G-IVF™ PLUS media (37 °C, 6% CO_2_ in air with maximum humidity; Vitrolife) and placed in four-well dishes (Nunc, Roskilde, Denmark) containing up to 500 µL of the same medium. Capacitated spermatozoa (after swim-up selection and 3-hour incubation) were added to each well at a final concentration of 200 000 or 400 000 sperm/mL. The beads and the spermatozoa were co-incubated overnight at 37 °C under 6% CO_2_ in air at 100% humidity.

Following overnight co-incubation with the beads, spermatozoa were stained with anti-CD46-FITC antibody M177 clone (3 µg/mL; Santa Cruz Biotechnology) for 30 minutes at 37 °C. After staining, unbound sperm were collected for flow cytometry (detailed below), meanwhile retaining the sperm–bead complexes in the well using magnets. Then, the sperm–bead complexes remaining in the wells were fixed with paraformaldehyde (2%) and counterstained with Hoechst 33342 (1 µg/mL; Sigma-Aldrich, Burlington, MA, USA) (30 minutes at RT). The stained and fixed sperm–bead complexes were transferred into a new well containing 0.5% (v/v) paraformaldehyde solution to remove loosely bound sperm and evaluated within 2 hours.

The percentage of beads with at least one sperm bound, the mean number of sperm bound to each bead, and its acrosome state (percentage of acrosome-reacted sperm among total bound sperm) were quantified using widefield fluorescence microscopy (Widefield Leica Thunder-TIRF imager, Leica Microsystems). A multidimensional acquisition approach was applied, including multichannel imaging, z-stack analysis, multiple stage positions, and mosaic imaging. The resulting images were analysed using an AI-based software (Leica Application Suite X (LAS X) version 3.10.0, Leica Microsystems). Fluorescent probes (FITC and Hoechst) were excited at 479 nm and 391 nm, respectively, with emissions detected at 519 nm and 435 nm, respectively. Acrosome-reacted cells exhibited green and blue fluorescence (CD46+ and Hoechst+), while cells with an intact acrosome displayed only blue fluorescence (CD46– and Hoechst+).

### Monoclonal antibodies inhibition assay

To evaluate the specificity of sperm binding to BJUNO via the IZUMO1–JUNO interaction, two monoclonal anti-IZUMO1 antibodies targeting two noncompeting epitopes were used (Tang *et al.*, 2022). Antibody 6F02 binds to the IZUMO1 epitope involved in JUNO recognition, thereby blocking the IZUMO1–JUNO interaction. In contrast, antibody 4E04 recognizes a different epitope not implicated in JUNO binding. Murine IgG1 was included as an isotype negative control. Antibodies were added directly into the wells immediately prior to sperm insemination, at two final concentrations: 2.5 µg/mL and 5 µg/mL.

### Flow cytometry

Acrosome integrity of in-suspension spermatozoa was assessed by flow cytometry just after thawing, after swim-up selection method, and before (3 hours in capacitation conditions after swim-up selection) and after co-incubation with the beads.

An aliquot of spermatozoa was mixed with Cell Staining Buffer (BioLegend, San Diego, CA, USA) and incubated with anti-CD46-FITC antibody M177 clone (3 µg/mL; Santa Cruz Biotechnology) for 30 minutes in the dark before evaluation.

Fluorescent probe (FITC) was excited with a 488 nm blue solid-state laser, and its emission was detected at 530 nm, using a BD FACSCanto flow cytometer (BD Biosciences, Milpitas, CA, USA). A total of 10 000 events were evaluated for each sample. Data acquisition and analysis were performed using BD FACSDiva software (BD Biosciences). Acrosome-reacted cells exhibited green fluorescence (CD46+).

### DNA integrity

DNA integrity was evaluated with the TUNEL assay using the In Situ Cell Death Detection Kit (Roche, Basel, Switzerland) according to Peris-Frau *et al.* (2022) for unbound spermatozoa. For bead-bound spermatozoa, some modifications were introduced. Briefly, following CD46–FITC staining and 2% (v/v) paraformaldehyde fixation, the beads were permeabilized with 0.1% (v/v) Triton X-100 in PBS for 5 minutes and washed with PBS. DNA fragmentation was detected by incubating each group of 30 beads with 15 μL of terminal deoxynucleotidyl transferase (TdT)-fluorescent-labelled (tetramethylrhodamine, TMR Red) nucleotide mix for 1 hour in a dark, humidified chamber at 37 °C. Then, they were washed with PBS and then counterstained with 0.1 mg/mL of Hoechst 33342 for 5 minutes to visualize total DNA. The sperm–bead complexes and the unbound spermatozoa were counted using widefield fluorescence microscopy (Widefield Leica Thunderxf-TIRF imager, Leica Microsystems). Fluorescent TMR Red probe was excited at 554 nm and detected at 594 nm, while FITC probe was excited at 450 nm and detected at 510 nm. Cells with fragmented DNA exhibited red fluorescence (TUNEL+), in addition to blue fluorescence (Hoechst+). In contrast, cells with intact DNA (TUNEL–) displayed only blue fluorescence (Hoechst+). Acrosome-reacted cells exhibited green and blue fluorescence (CD46+ and Hoechst+).

### Hamster IVF

Hamster oocytes (Janvier Labs, Le Genest-Saint-Isle, France) were thawed according to the supplier’s instructions and subsequently treated with 1 mg/mL Tyrode’s acid solution to remove the ZP, yielding zona-free oocytes. Each zona-free oocyte was incubated overnight with 20 000 spermatozoa in FertiCult medium supplemented with 3% (w/v) BSA (FertiPro) at 37 °C in an atmosphere of 6% CO_2_ with high humidity. Following incubation, sperm–oocyte complexes were then washed briefly to remove loosely bound sperm, fixed in 2% (w/v) paraformaldehyde, and counterstained with Hoechst 33342 (1 µg/mL; Sigma-Aldrich) for 30 minutes at RT. Fluorescence imaging was performed using a widefield fluorescence microscope (Leica Thunder Widefield/TIRF system, Leica Microsystems). A multidimensional acquisition approach was applied, including multichannel imaging, z-stack analysis and mosaic imaging to evaluate number of human sperm bound per zona-free hamster egg and sperm head decondensation.

### Statistical analysis

Data were analysed using RStudio (R version 4.1.3; Posit PBC, Boston, MA, USA) (RStudio Team, 2020). For the, presented as mean ± SEM, a generalized linear mixed-effects model (GLMM) for negative binomial family was applied given the distribution of the data. Pairwise contrasts were performed to compare groups using the Benjamini–Hochberg procedure (false discovery rate, FDR).

For the results presented as percentages ± 95% CI (beads with at least one sperm bound, acrosome reaction and DNA integrity), a logistic regression for binomial data (mixed-effects model, GLMM) was used to evaluate the independence between variables. Pairwise comparisons of proportions were conducted with FDR correction.

For the classification of semen donors, an unsupervised cluster analysis was performed using the *k-means* algorithm. Donors were clustered into two groups (*k *= 2) based on the mean number of sperm bound per bead across replicates and the t-score (mean/SEM) for each donor, after standardizing and reweighting the variables by multiplying them by 1 and 0.5, respectively. Subsequently, a ROC curve analysis was conducted to determine the optimal cut-off value.

## Results

### Human fresh spermatozoa bind specifically to JUNO-coated beads

**Figure 1.**
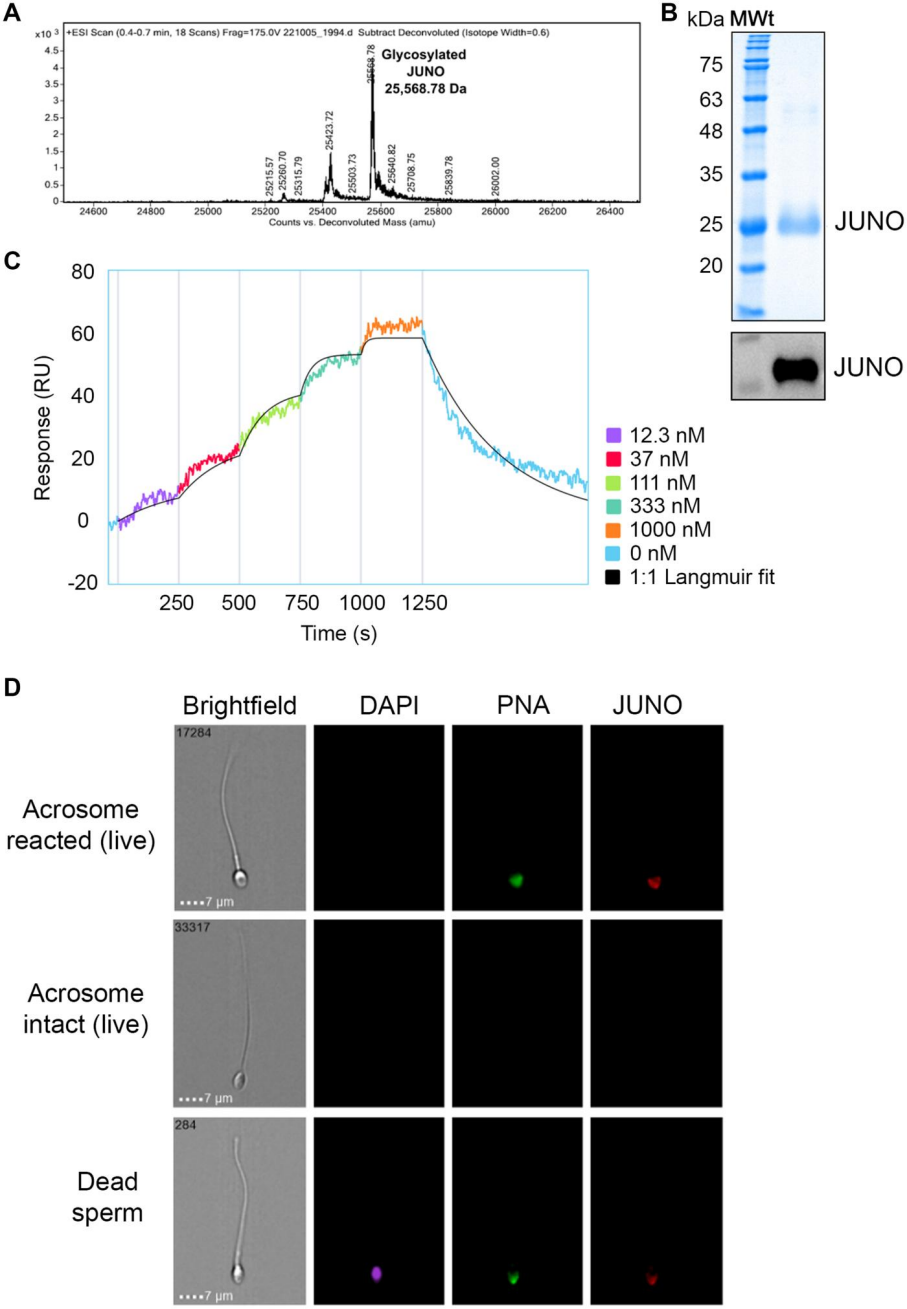
**Recombinant human JUNO binds to acrosome-reacted human sperm.** (**A**) Electrospray ionization–mass spectrometry (ESI–MS) of human His-tagged glycosylated JUNO (residues 20–228) shows a peak corresponding to the expected mass (25 568.8 Da). (**B**) Coomassie-stained SDS–PAGE and anti-His western blot analyses of purified, glycosylated JUNO reveal a ∼26 kDa band, consistent with its predicted molecular weight. (**C**) Digital surface plasmon resonance demonstrates binding between human JUNO and human IZUMO1 ectodomain, with KD = 37.6 ± 0.9 nM, k_on_ = (1.03 ± 0.09) × 10^5^ M^−1^ s^−1^ and k_off_ = (3.87 ± 0.36) × 10^−3^ s^−1^ (*n* = 3) (**D**) Imaging flow cytometry of fluorophore-labeled, acrosome-reacted human sperm confirms JUNO binding.

Intact-protein ESI–MS, Coomassie-stained SDS–PAGE and anti-His Western blot analyses confirmed the purity and identity of the expressed and purified JUNO protein used in the bead-based assay (Fig. 1A and B). Digital SPR demonstrated that recombinant JUNO binds the human IZUMO1 ectodomain with a KD of 38 nM, consistent with previously reported affinities (Aydin *et al.*, 2016) (Fig. 1C). The biological activity of recombinant JUNO was validated by assessing its ability to recognize human sperm at different acrosomal states: imaging flow cytometry showed specific binding to acrosome-reacted sperm, but not to acrosome-intact sperm (Fig. 1D).

**Figure 2.**
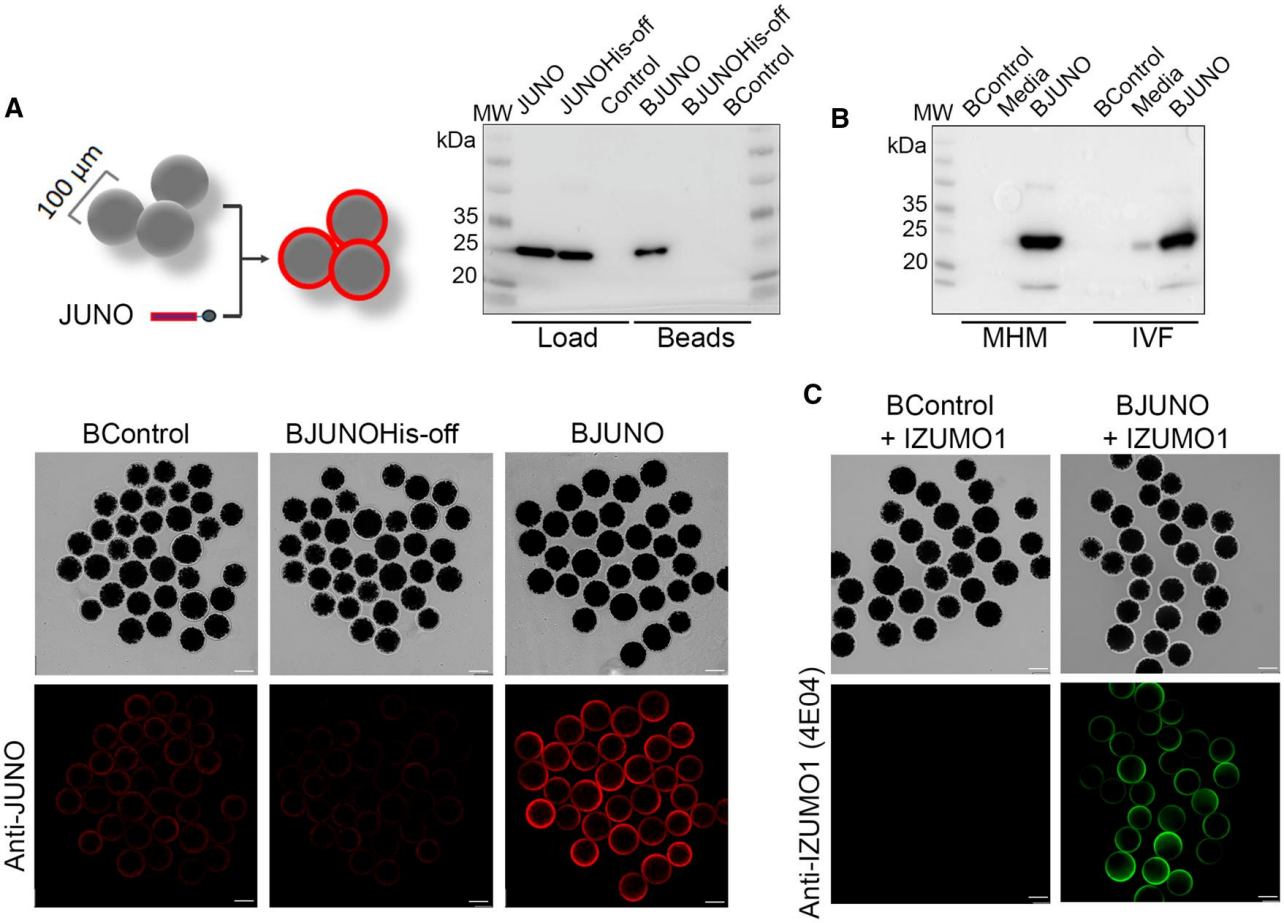
**Recombinant human JUNO protein stably conjugated to beads preserves IZUMO1 binding activity.** (**A**) Schematic representation and validation of JUNO–bead conjugation by anti-JUNO Western blot and immunodetection. Load lanes correspond to 0.3 µg of His-tagged (JUNO) and untagged (JUNOHis-off) recombinant JUNO proteins; blank buffer control (Control). Eluate lanes show the protein detected after elution from 60 beads incubated with JUNO (BJUNO), JUNOHis-off (BJUNOHis-off), or without protein (BControl). A clear band is observed in beads conjugated with JUNO. Widefield fluorescence images show a uniform JUNO coating on BJUNO bead surfaces, absent in BControl and BJUNOHis-off. Scale bar, 50 µm. (**B**) Stability of JUNO conjugation after overnight (ON) incubation was assessed using two different media. BControl and BJUNO lanes represent eluates from beads of each group, and the media lanes correspond to BJUNO supernatants after ON incubation in MHM-C (lanes 1–3) or IVF-PLUS (lanes 4–6). No JUNO protein was detected in BJUNO supernatants from either medium, indicating stable conjugation after overnight incubation at 37 °C. (**C**) Specific binding of recombinant IZUMO1 to JUNO-beads was evaluated by anti-IZUMO1 (4E04) immunodetection. Widefield fluorescence images show specific IZUMO labelling on the surface of JUNO-beads (BJUNO). Scale bar, 50 µm.

His-tagged recombinant human JUNO protein was successfully conjugated to the Sepharose beads and a uniform coating on the JUNO-beads surface was observed by widefield fluorescence microscopy revealed with anti-JUNO antibody (Fig. 2A; Supplementary Fig. S1). To evaluate the stability of the JUNO protein conjugated to the beads under IVF assay conditions, JUNO-beads were incubated overnight at 37 °C, 6% CO_2_ in air with maximum humidity. Two different IVF media commonly used in assisted reproduction technologies were tested: Multipurpose Handling Medium-Complete (MHM-C, FUJIFILM Irvine Scientific, Inc., USA), and G-IVF™ PLUS (Vitrolife) (Fig. 2B). This assay demonstrated that the rhJUNO protein remains stably conjugated to the beads under standard IVF incubation conditions. Furthermore, the biological activity of bead-conjugated JUNO was validated through the specific binding of human recombinant IZUMO1 to JUNO-beads (Fig. 2C).

**Figure 3.**
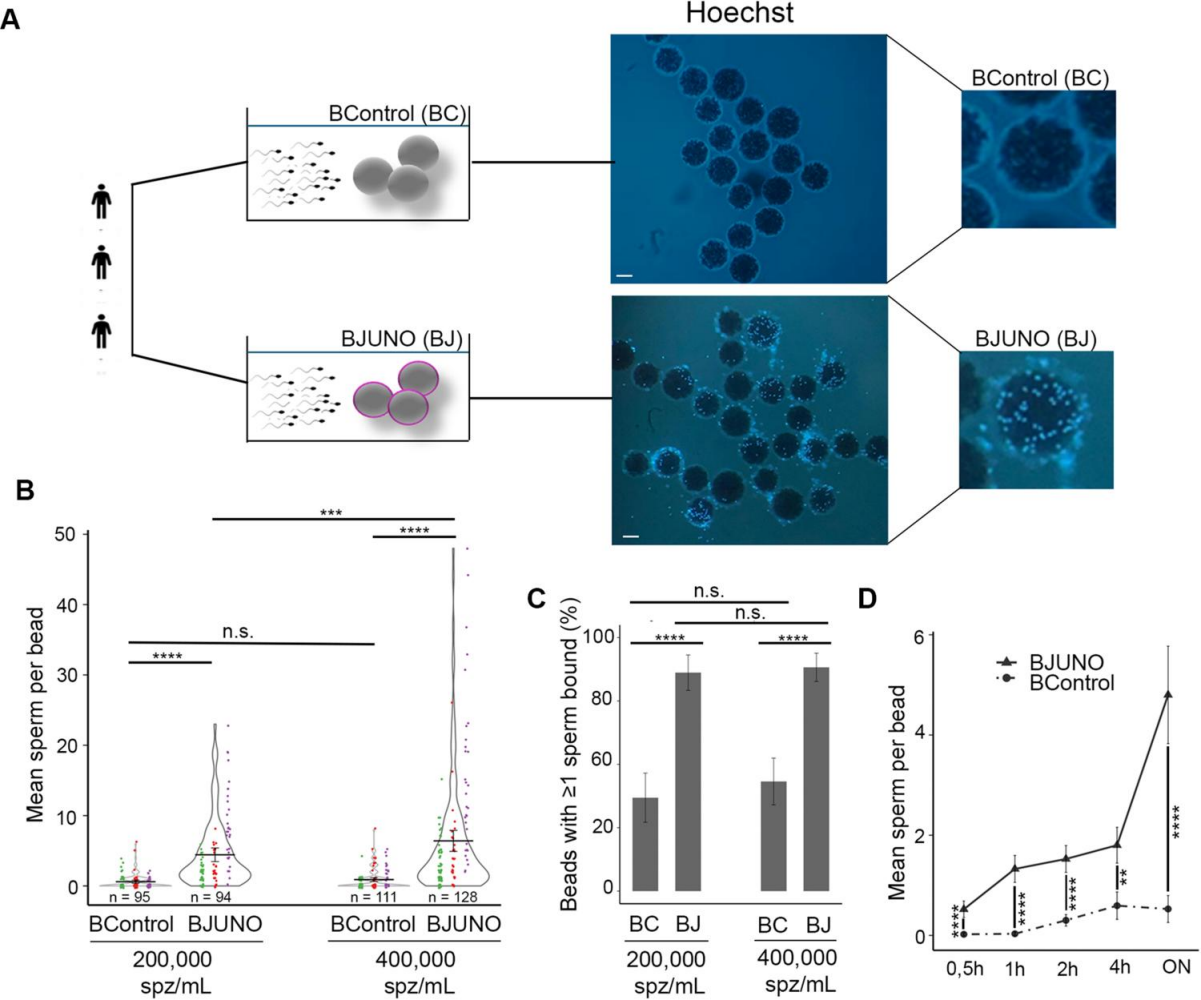
**Sperm-binding assay to JUNO-beads (BJUNO) using human fresh semen samples.** (**A**) Schematic and epifluorescence microscopy images (Hoechst) of BControl (BC) and BJUNO (BJ) after co-incubation with spermatozoa from three different donors. Scale bar, 100 µm. (**B**) Quantification of sperm–bead interaction under two sperm concentrations (200 000 and 400 000 spermatozoa/mL). Only spermatozoa whose heads were in contact with the bead were counted. Mean number of spermatozoa per bead (violin plot, mean ± S.E.M.; *n* = 3). Circles indicate individual data points from three donors coloured green, red, and blue; bars represent mean. (**C**) Percentage of beads with at least one bound sperm (bar plot, percentage ± 95% CI; *n *= 3). Both parameters were significantly higher for BJUNO compared to BControl. (**D**) Time-course analysis (line plot, mean ± S.E.M., *n *= 3) of sperm-binding dynamics, showing a progressive increase in sperm attachment to BJUNO over time, with overnight (ON) incubation yielding the highest number of sperm per bead. n.s., not significant; *P < 0.05; **P ≤ 0.01; ***P ≤ 0.001; ****P ≤ 0.0001.

To assess human sperm-binding to JUNO-beads, freshly ejaculated sperm samples from three different donors were co-incubated with JUNO-beads (BJUNO) and Control-beads (BControl) at two sperm concentrations comparable to those used in IVF protocols. After the incubation period commonly used in IVF procedures, sperm bound to the beads (identified as Hoechst-positive blue dots) (Fig. 3A) were quantified to evaluate two key parameters of binding performance for each sample: (i) the average number of sperm cells attached per bead, and (ii) the proportion of beads with at least one attached sperm cell, as a measure of binding specificity. Evaluation under a fluorescent microscope revealed that the BJUNO group had a significantly higher number of bound sperm cells compared with the BControl group (*P *≤ 0.0001). Remarkably, increasing the concentration led to a 150% rise in the number of attached sperm cells in BJUNO, from 4.4 ± 0.5 to 6.4 ± 0.8 (*P *< 0.001). In contrast, this increase had no significant effect on the control group, where the number of bound sperm cells remained relatively unchanged (0.6 ± 0.1 and 0.9 ± 0.1, respectively; *P *= 0.056) (Fig. 3B). When evaluating the percentage of beads with at least one bound sperm at both sperm concentrations, we observed that the majority of BJUNO had sperm attached (88.3%, CI 95%: 82.7–93.9%, *n* = 128 beads), whereas in the control group, most beads showed no bound sperm (43.2%, CI 95%: 34.0–52.5%, *n* = 111 beads) (*P *< 0.0001) (Fig. 3C). Taken together, these results indicate that human sperm bind specifically and, in a dose-dependent manner to BJUNO, and that this binding capacity to the bead model is dependent on the presence of the receptor JUNO.

We subsequently selected a dose of 400 000 spermatozoa/mL to maximize the number of bound sperm and, consequently, increase the sensitivity of the assay. To determine the optimal incubation time, binding kinetics were assessed at 30 minutes, 1 hour, 2 hours, 4 hours, and overnight. Sperm binding to BJUNO increased progressively, with significant differences from BControl evident as early as 30 minutes (*P *< 0.0001). The greatest difference was observed after overnight incubation, with an average of 4.8 ± 0.5 sperm bound per bead in the BJUNO group (*n* = 90 beads) versus 0.5 ± 0.1 in the BControl group (*n* = 91 beads) (*P *< 0.0001) (Fig. 3D; Supplementary Table S3). A similar trend was observed when analysing the percentage of beads with at least one bound sperm (Supplementary Table S3). These results indicate that the specific binding of human sperm to BJUNO is also time dependent.

### Vitrified human sperm bind specifically to JUNO beads

**Figure 4.**
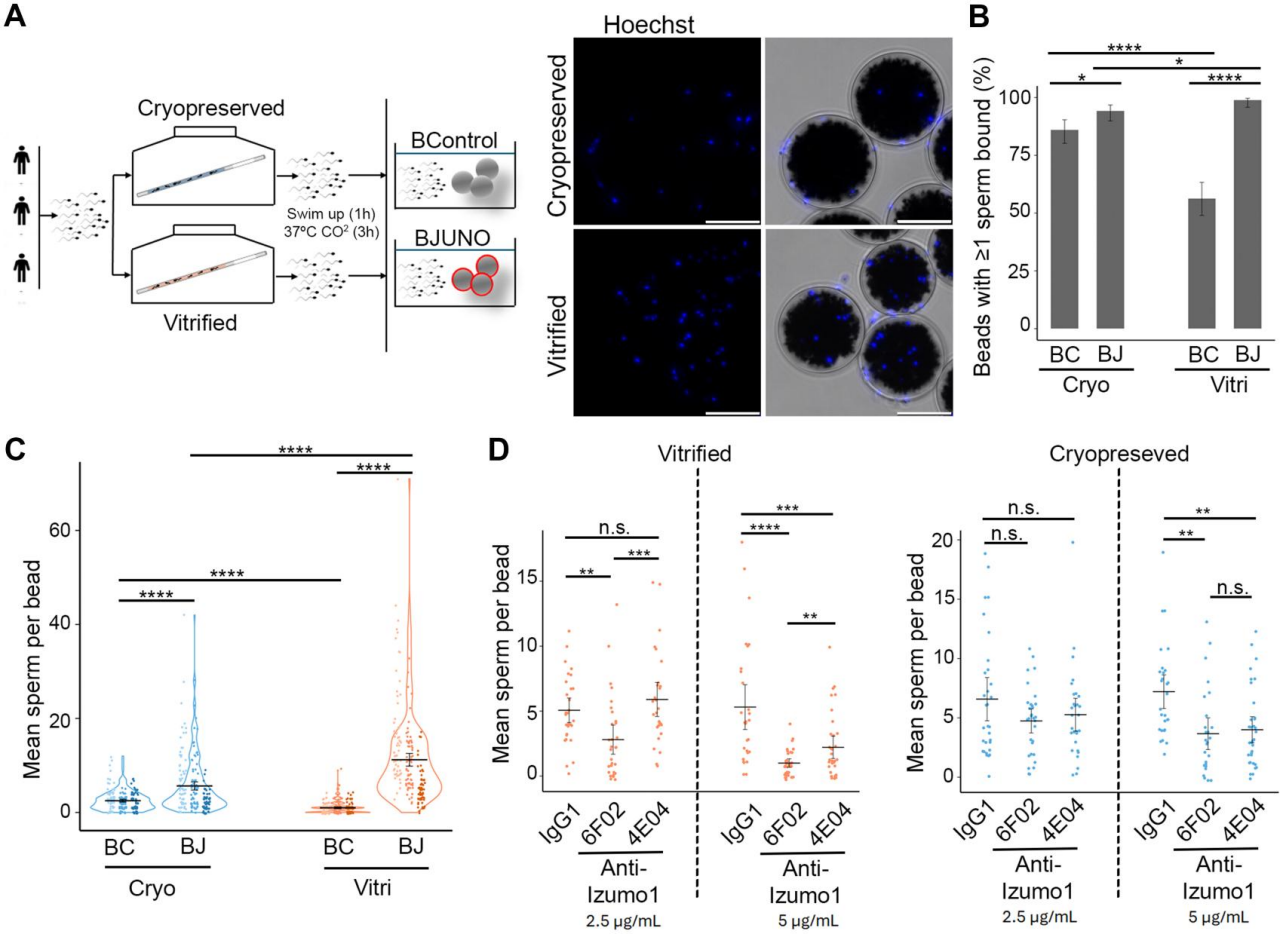
**Effect of freezing method on sperm binding to JUNO-beads.** (**A**) Schematic representation of the experimental groups (cryopreserved vs. vitrified) (left panel) and the widefield fluorescence representative images (Hoechst) of the sperm-binding assay (right panel) on the BJUNO. Only spermatozoa with their heads in contact with the bead were counted. Scale bar, 100 µm. (**B**) The percentage of beads with at least one bound sperm was quantified for both cryopreserved and vitrified samples (bar plot, percentage ± 95% CI; *n* = 3). BJUNO (BJ) showed a consistently higher binding compared to BControl (BC) in both types of preserved samples. (**C**) The mean number of spermatozoa bound per bead was compared between BJ and BC for each preservation method (violin plot, mean ± S.E.M.; *n* = 3). Circles indicate individual data points from three donors coloured by grade intensity, bars represent the mean. A significantly higher binding was observed for BJUNO, with the difference between BJ and BC being more pronounced in vitrified samples. (**D**) Specificity of the sperm-binding assay was tested using anti-IZUMO1 antibodies (4E04 and 6F02) at two concentrations (2.5 µg/mL, left; and 5 µg/mL, right). Individual data points are shown; bars represent the mean ± S.E.M. These antibodies target different epitopes of IZUMO1, with 6F02 specifically blocking the epitope involved in JUNO binding. IgG1 was used as a negative antibody control. In cryopreserved samples, no significant difference was observed at 2.5 µg/mL, while both antibodies reduced sperm binding at 5 µg/mL. In vitrified samples, 6F02 caused a significant reduction in sperm binding at both concentrations, with stronger inhibition at 5 µg/mL. n.s., not significant; *P < 0.05; **P ≤ 0.01; ***P ≤ 0.001; ****P ≤ 0.0001.

To evaluate the model, we compared the binding capacity of sperm samples subjected to two different cryopreserved methods, based on the premise that such procedures can affect membrane stability (Barthelemy *et al.*, 1990) and, consequently, the presentation of receptors involved in sperm–oocyte binding. For this analysis, ejaculates from three donors were split into two aliquots: one processed using a cryopreserved-based method, while the other was frozen by vitrification (Fig. 4A, Supplementary Table S4). After thawing, samples (400 000 sperm/mL) were incubated overnight with BJUNO and BControl, and analysed as previously described.

We observed that vitrified samples exhibited a significantly higher proportion of BJUNO with at least one bound sperm (98.9%, 95% CI: 95.8–99.7%) compared to BControl (56.3%, 95% CI: 49.0–63.3%; *P *≤ 0.0001), consistent with the results obtained from fresh samples. In cryopreserved samples, although a statistically significant difference was still observed between BJUNO (94.2%, 95% CI: 89.9–96.8%) and BControl (86.0%, 95% CI: 80.3–90.3%; *P *< 0.05), the magnitude of the difference was smaller. Moreover, in the cryopreserved group, a larger proportion of BControl presented at least one bound sperm, suggesting a higher number of sperm undergoing non-specific binding (Fig. 4B).

Regarding the average number of sperm bound per bead (Fig. 4C), vitrified samples again showed superior binding efficiency, with BJUNO displaying 11.2 ± 0.7 bound spermatozoa (*n* = 188 beads) compared to 0.98 ± 0.1 in BControl (*n* = 183 beads). In cryopreserved samples, binding was lower overall, with 5.6 ± 0.4 sperm bound to BJUNO (*n* = 191 beads) and 2.5 ± 0.2 to BControl (*n* = 186 beads). While both methods demonstrated preferential binding to BJUNO over BControl (*P *≤ 0.0001), sperm from vitrified samples exhibited significantly greater binding efficiency compare with sperm from slow freezing (*P *≤ 0.0001). Additionally, the number of sperm bound to BControl was significantly lower in vitrified samples compared to cryopreserved ones (*P *≤ 0.0001), further supporting that the vitrified method better preserves the cellular and molecular conditions necessary for specific binding to BJUNO.

To demonstrate that human sperm binding to BJUNO is specifically mediated by the IZUMO1–JUNO interaction, two well-characterized anti-IZUMO1 antibodies targeting distinct epitopes were used (Fig. 4D). Anti-IZUMO1 6F02 blocks the epitope directly involved in IZUMO1–JUNO binding, whereas anti-IZUMO1 4E04 recognizes a different region not implicated in this interaction (Tang *et al.*, 2022). In vitrified samples (Fig. 4D, left graph), the use of anti-IZUMO1 6F02 at 2.5 µg/mL significantly reduced sperm binding compared to the control (2.8 ± 0.6 vs. 5.1 ± 0.5; *P *< 0.01), confirming specific inhibition of the IZUMO1-mediated interaction. In contrast, anti-IZUMO1 4E04 had no significant effect at the same concentration (5.9 ± 0.7; *P *> 0.05). When the antibody concentration was increased to 5 µg/mL, both antibodies reduced sperm binding, but the effect was significantly stronger with anti-IZUMO1 6F02 (1.0 ± 0.2 vs. 5.3 ± 0.9; *P *< 0.0001).

In cryopreserved samples (Fig. 4D, right graph), the inhibitory effect of the antibodies was less pronounced. At 2.5 µg/mL, neither anti-IZUMO1 6F02 nor 4E04 significantly reduced sperm binding compared to the control (4.7 ± 0.5, 5.3 ± 0.7, and 6.6 ± 0.9, respectively; *P *> 0.05). At 5 µg/mL, both antibodies caused a moderate but comparable reduction (3.7 ± 0.7, 4.0 ± 0.6, and 7.2 ± 0.7, respectively; *P *< 0.01), suggesting that in these samples, in addition to the specific IZUMO1–JUNO interaction, nonspecific binding mechanisms may also contribute.

**Figure 5.**
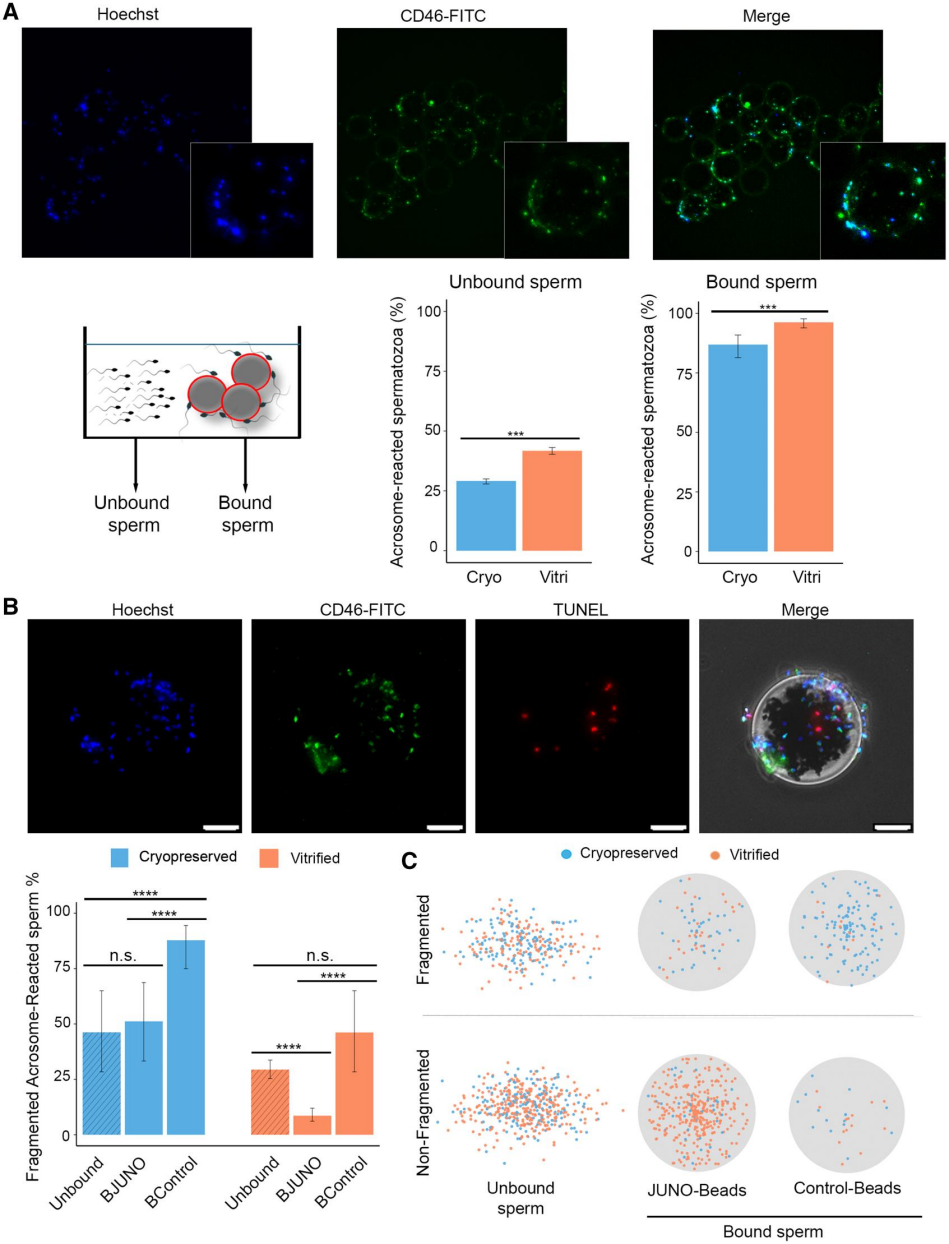
**Acrosome reaction and DNA fragmentation evaluation on sperm–JUNO bead binding assay.** (**A**) Wide-field fluorescence representative images of the spermatozoa (Hoechst) and acrosome-reacted spermatozoa (CD46–FITC) bound to JUNO-beads. The percentage of acrosome-reacted spermatozoa (Hoechst+ and CD46+) was quantified in unbound sperm by flow cytometry (left bar plot, percentage ± 95% CI; *n* = 3) and BJUNO-bound sperms by widefield fluorescence (right bar plot, percentage ± 95% CI, *n* = 3), revealing a ∼50% enrichment of acrosome-reacted spermatozoa in the BJUNO group across both types of preserved samples. (**B**) DNA integrity of acrosome-reacted spermatozoa was assessed by TUNEL assay (widefield fluorescence) in both bead-bound and unbound cells (upper panels). Scale bar, 10 µm. The percentage of acrosome-reacted spermatozoa with DNA fragmentation (left bar plot, percentage ± 95% CI, *n* = 3) was higher in cryopreserved samples than in vitrified ones. Additionally, BJUNO-bound spermatozoa showed lower levels of DNA fragmentation compared to BControl-bound and unbound sperm. (**C**) The individual cell (spermatozoa) distribution according to DNA integrity of acrosome-reacted spermatozoa evaluation is represented in the right dot plots (dots indicate individual spermatozoa). n.s., not significant; *P < 0.05; **P ≤ 0.01; ***P ≤ 0.001; ****P ≤ 0.0001.

A key cellular requirement for gamete interaction is that spermatozoa must be acrosome-reacted. Loss of the acrosome leads to the localization of IZUMO1 at the equatorial segment of the sperm head, enabling its interaction with JUNO present on the oocyte membrane (Satouh *et al.*, 2012; Inoue and Wada, 2018; Manjon *et al.*, 2025). Therefore, sperm that bind specifically to JUNO-coated beads are expected to be acrosome-reacted. To investigate this, we first assessed by flow cytometry the acrosomal status of cryopreserved and vitrified sperm samples using the same incubation times as in the sperm–beads binding assay, but without the beads (Supplementary Fig. S2), using CD46 as a marker for acrosome-reacted live spermatozoa (CD46+). The most significant difference was observed after overnight incubation, where vitrified samples showed a significantly higher percentage of acrosome-reacted cells (19.2%) compared to cryopreserved samples (10.0%) (*P *< 0.0001), as determined by flow cytometry. After establishing the acrosomal status under our experimental conditions, we then evaluated the acrosomal status of spermatozoa in the sperm–bead binding assay after overnight incubation, comparing sperm bound to the beads *versus* those remaining unbound. In the unbound population (Fig. 5A), the proportion of acrosome-reacted spermatozoa was relatively low, 29.1% (95% CI: 27.9–30.0%) in cryopreserved and 41.7% (95% CI: 40.3–43.1%) in vitrified samples (Fig. 5A, unbound sperm graph). In contrast, most of sperm bound to the beads were acrosome-reacted, with over 85% being CD46+ in both conditions (86.9%, 95% CI: 81.4–90.9% for cryopreserved and 96.2%, 95% CI: 93.9–97.7% for vitrified samples) (Fig. 5A, bound sperm graph), indicating that binding to JUNO-coated beads selectively occurs in acrosome-reacted spermatozoa.

The acrosome reaction could be associated with, and may be both a cause and consequence of, sperm cell death (Harper *et al.*, 2008). To determine whether sperm bound to JUNO-coated beads undergo acrosome reaction as a functional requirement for receptor interaction, rather than as a degenerative event, we evaluated DNA integrity in acrosome-reacted sperm using the TUNEL assay (Fig. 5B). In vitrified samples, 29.4% (95% CI: 25.4–33.7%) of unbound reacted spermatozoa exhibited DNA fragmentation. In contrast, reacted spermatozoa bound to JUNO beads showed a significantly lower rate of DNA fragmentation (8.6%; 95% CI: 6.1–12.0%; *P *< 0.0001) (Fig. 5B, orange graph), indicating that most JUNO-bound sperm are acrosome-reacted and maintain intact DNA, a hallmark of functional viability. This contrasts with the BControl-bound group, in which 46.2% (95% CI: 28.4–65.0) of acrosome-reacted spermatozoa exhibited DNA fragmentation (*P *> 0.05 vs. unbound), suggesting that damaged sperm may bind non-specifically (Fig. 5B, orange graph). A similar pattern was observed in cryopreserved semen samples (Fig. 5B, blue graph), where 51.2% (95% CI: 33.3–68.7) of unbound reacted spermatozoa showed DNA fragmentation, and a comparable percentage was found among BJUNO-bound spermatozoa (46.0%; 95% CI: 28.1–64.9; *P *> 0.05). In contrast, the Control-bound fraction displayed significantly higher DNA fragmentation (87.8%; 95% CI: 75.0–94.5; *P* < 0.0001 vs. unbound) (Fig. 5B, blue graph). These results suggest that cryopreserved samples bind more non-specifically to surfaces, likely due to increased cellular damage.

When acrosome-reacted spermatozoa were further classified based on the presence or absence of DNA fragmentation, represented by individual sperm counts (dots) across assay conditions for unbound, JUNO-beads and control beads, a clear pattern emerged (Fig. 5C). The population of non-fragmented, acrosome-reacted spermatozoa was most enriched among vitrified samples bound to JUNO-beads, whereas in cryopreserved samples, a greater number of DNA fragmented spermatozoa were bound to control beads. These findings indicate that cryopreserved samples not only show reduced binding to JUNO-beads, but also exhibit increased non-specific binding, likely as a result of greater cellular damage. This is supported by the observed DNA fragmentation, which may be linked to membrane destabilization caused by the freezing process, potentially leading to mislocalization of the IZUMO1-binding complex.

Together, these results demonstrate that JUNO-coated beads selectively bind vitrified spermatozoa exhibiting features essential for fertilization, namely, acrosome-reacted status and low DNA fragmentation, underscoring the specificity and biological relevance of the assay.

### Sperm–bead binding assay as a functional evaluation of semen quality

**Figure 6.**
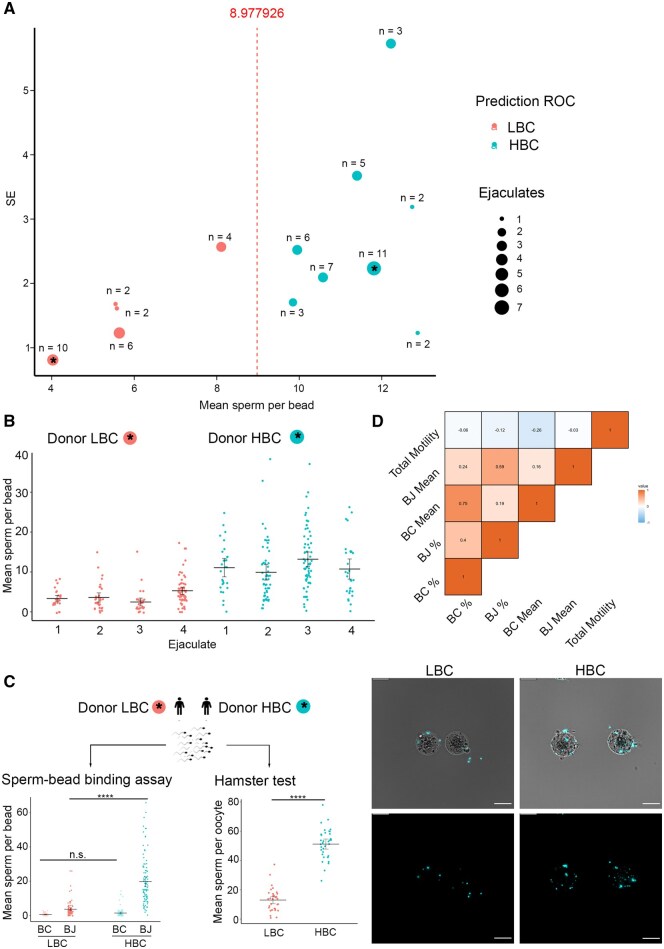
**Validation of the sperm–bead binding assay.** (**A**) Representation of semen donors’ classification based on the mean number of sperm bound per bead and its standard error (SE) across replicates. Dot size reflects the number of different ejaculates per donor, and the number next to each dot indicates the number of replicates. Donors classified as low-binding capacity (LBC) are shown in red, and those classified as high-binding capacity (HBC) are shown in blue. (**B**) Mean number of spermatozoa bound per bead from two donors previously classified as LBC (red dots) and HBC (blue dots), using four ejaculates collected at different time points. Circles indicate individual data points (JUNO-Beads), bars represent mean ± S.E.M. (**C**) Human semen samples from LBC (red dots) and HBC (blue dots) donors were split and evaluated using both the hamster penetration test and the sperm–beads binding assay. The mean number of spermatozoa bound per bead was compared between each donor (left dot plot, mean ± S.E.M.; *n* = 3). Circles indicate individual data points (JUNO-Beads) from three replicates of the same ejaculate for each donor; bars represent the mean. The HBC donor showed a significantly higher number of sperm bound to JUNO beads (BJ) (19.78 ± 1.43, *n* = 98; *P *< 0.0001) than the LBC donor (3.60 ± 0.46, *n *= 96). The summary of the number of human sperm bound per zona-free hamster oocyte (right dots plot, mean ± SEM, *n* = 3) was compared between LBC (red dots) and HBC (blue dots) donors. Circles represent individual data points (hamster oocytes) from three replicates of the same ejaculate for each donor; bars represent the mean. The HBC donor showed a significantly higher number of sperm bound to zona-free hamster oocytes (51.17 ± 1.76, *n* = 35; *P *< 0.0001) than the LBC donor (12.97 ± 1.27, *n* = 36). Representative images show the binding of human sperm (Hoechst) from LBC and HBC to zona-free hamster oocytes. (**D**) Pearson correlation analysis between total sperm motility prior to co-incubation (total motility) and the two main readouts of the sperm-binding assay: the mean number of spermatozoa bound per bead (BJ Mean and BC Mean) and the percentage of beads with at least one sperm bound (BJ % and BC %). Weak linear relationships were observed between total motility and the assay readouts. Abbreviation: n.s., not significant; ****P ≤ 0.0001. Scale bar, 50 µm.

Once we confirmed that vitrified sperm bound to JUNO-beads exhibit the hallmark features of fertilization-competent sperm and that their binding can be quantitatively measured, we compared binding capacity across normalized semen samples from different donors. This enables assessment of inter-individual variability and supports the potential utility of the sperm–bead binding assay as a functional test for sperm quality. Using vitrified sperm from 18 individual donors (29 ejaculates and 50 replicates) and analysing over 3300 beads, we observed that the mean number of spermatozoa bound to JUNO beads was significantly higher than that bound to Control beads (8.2 ± 0.2, *n* = 1679 *vs*. 0.7 ± 0.0, *n* = 1689; *P *< 0.0001) (Supplementary Fig. S3; Supplementary Table S5). To stratify donors based on sperm–bead binding performance, we performed unsupervised k-means clustering on donors (*n* = 13) with at least two technical replicates using two features; the mean number of sperm bound per bead and the t-score (mean/SE) across replicates (Fig. 6A). The k-means algorithm grouped donors into two clusters: five donors with low-binding capacity (LBC), and eight with high-binding capacity (HBC). A ROC curve was subsequently generated using the mean sperm count per bead as a discriminating variable, identifying an optimal cut-off value of 9.00 spermatozoa per bead (Fig. 6A). To illustrate intra-donor variability, we highlight the profiles of two donors, one classified as LBC and the other as HBC (Fig. 6B). We further validated the bead assay by evaluating semen samples from the two selected donors using both the sperm–bead binding assay and the hamster oocyte penetration test (Fig. 6C), which showed that semen samples from the LBC-classified donor bound significantly fewer sperm (12.97 ± 1.27, *n* = 36; *P *< 0.0001) to zona-free hamster oocytes compared with those from the HBC-classified donor (51.17 ± 1.76, *n* = 35) (Fig. 6C, Supplementary Fig. S4). Together, these results demonstrate that the sperm–bead binding assay can classify individuals based on the binding capacity of their semen samples to the oocyte membrane. Ultimately, clinical studies correlating bead-binding performance with assisted reproduction outcomes (e.g. insemination or IVF) will be needed to determine the predictive value of the assay.

Finally, we assessed whether the mean number of spermatozoa bound per bead or the percentage of beads with at least one sperm bound were linearly associated with total sperm motility prior to co-incubation (Fig. 6D). Pearson correlation analyses revealed very weak associations between motility and all binding parameters (|r| < 0.27), indicating a negligible or absent linear relationship.

## Discussion

The model presented builds upon a previously described and validated system tested in other species using recombinant proteins involved in gamete interaction (Hamze *et al.*, 2020a,b,c). In the present study, we adapted the 3D-bead-based model for human application using recombinant human JUNO protein that specifically binds to human acrosome reacted sperm. The approach is designed to model key aspects of IVF by replacing the oocyte with JUNO-coated beads that serve as molecular decoys, enabling the selection of competent sperm capable of specifically binding to JUNO. To establish proof of concept, the experimental conditions, such as sperm concentration, incubation time, and media, were aligned with standard human IVF protocols.

Our findings reveal a clear time- and concentration-dependent pattern of sperm binding specifically to JUNO-coated beads, consistent with the highly mechanostable, multistate catch-bond mechanism recently described for the IZUMO1:JUNO interaction (Boult *et al.*, 2025). This mechanism enables stable long-term adhesion between the sperm and oocyte and was also observed using the JUNO-bead model. Both the number of spermatozoa attached per bead and the proportion of beads with at least one bound sperm were significantly higher for JUNO-beads compared to control beads. This strong signal-to-noise ratio stands in sharp contrast to findings in bovine samples using the same approach (Hamze *et al.*, 2020c), where no significant differences were observed in the proportion of beads with at least one bound sperm. This species-specific disparity underscores that, under the tested conditions, human sperm exhibit a markedly higher efficiency in binding to JUNO-coated beads. These results also emphasize that both metrics, the number of sperm per bead and the proportion of beads with at least one bound sperm, are essential for a comprehensive interpretation of assay performance. Remarkably, efficient binding to JUNO-beads was achieved with substantially fewer spermatozoa (∼6000–7000 per bead) compared to the typical sperm-to-oocyte ratios used in IVF (∼50 000) (Oseguera-López *et al.*, 2019). More than 90% of JUNO-beads captured at least one sperm, enabling bead-by-bead evaluation within groups and greatly increasing the statistical robustness, and therefore the sensitivity, of the assay.

The preservation of gametes and embryos has been a cornerstone advance in ARTs, ensuring a reliable source of high-quality samples for both clinical and research applications. Vitrification of oocytes and embryos, in particular, has proven highly effective because it minimizes membrane damage and preserves cellular integrity and viability (Valojerdi and Salehnia, 2005; Rienzi *et al.*, 2017). Extending this strategy to sperm, vitrification offers the potential to maintain optimal cellular conditions, thereby improving the functional competence of preserved spermatozoa.

The novelty of the present study lies in the evaluation of JUNO-binding capacity in spermatozoa subjected to different preservation protocols. Our findings reveal that vitrified sperm retain a higher capacity for specific binding to JUNO-beads compared to standard cryopreservation. In contrast, sperm samples subjected to slow-rate cryopreservation exhibited a pronounced increase in non-specific binding, indicating that vitrification preserves the specific functionality of IZUMO1 required for fertilization-competent interactions, IZUMO1:JUNO.

Recent studies have described a complex molecular architecture underlying gamete recognition, in which IZUMO1 is part of a major sperm membrane protein complex (Ellerman *et al.*, 2009; Contreras *et al.*, 2022; Lu *et al.*, 2023; Pacak *et al.*, 2023) where IZUMO1, SPACA6, and TMEM81 specifically interact to form a functional complex (Deneke *et al.*, 2024). This trimeric complex interacts with the oocyte proteins JUNO and CD9, forming a hetero-pentameric structure at the fertilization synapse (Elofsson *et al.*, 2024). Specific monoclonal antibodies, 4E04 and 6F02, both recognize IZUMO1 at non-competing epitopes: notably, the 6F02 epitope overlaps with the IZUMO1-binding site for JUNO (Tang *et al.*, 2022). Consistent with the requirement to assemble this molecular structure to execute sperm–oocyte binding, in vitrified sperm samples, we observed that binding was inhibited by 6F02 but not by 4E04, except at high concentrations where partial inhibition likely reflects steric hindrance. In contrast, this inhibitory effect was absent in cryopreserved sperm, suggesting that the molecular organization required for specific JUNO-mediated binding is disrupted under these preservation conditions.

To further characterize JUNO bead-bound sperm, we evaluated their acrosomal status and DNA integrity. These analyses demonstrated that JUNO-coated beads selectively capture vitrified spermatozoa exhibiting hallmarks of fertilization competence, specifically, acrosome-reacted cells with minimal DNA fragmentation (Santi *et al.*, 2018; Tanga *et al.*, 2021; Morohoshi *et al.*, 2023). In contrast, although a large proportion of cryopreserved spermatozoa also underwent the acrosome reaction, a significant fraction displayed DNA fragmentation. This pattern suggests that slow freezing compromise membrane integrity, leading to premature loss of acrosomal contents and triggering apoptotic pathways associated with DNA damage (Duru *et al.*, 2001; Ozkavukcu *et al.*, 2008).

Together, these results indicate that cryopreservation compromises the specificity of IZUMO1–JUNO interactions by inducing membrane damage (Ozkavukcu *et al.*, 2008) that disrupts the spatial organization of receptors (Wang *et al.*, 2014; Fukuda *et al.*, 2016; Peris-Frau *et al.*, 2020). This loss of structural integrity increases the contribution of non-specific interactions, in sharp contrast to vitrified sperm, which maintains a molecular architecture conducive to selective and functional binding. The application of sperm vitrification in IVF techniques may represent an improvement in sperm quality with respect to fertilizing capacity, which could in turn contribute to enhanced embryo viability and development. Finally, we classified all donor samples evaluated in this study according to their JUNO-bead binding capacity, following the same approach previously applied to ejaculated frozen–thawed sperm from Holstein bulls with high and low fertility. As reported earlier (Hamze *et al.*, 2020c), sperm from low-fertility bulls exhibited a markedly reduced ability to bind JUNO-beads compared with high-fertility bulls, whose fertility had been confirmed through field performance and IVF cleavage rates. Although this approach requires further validation, since sperm binding to JUNO is not sufficient for fertilization to occur, our results highlight the potential of this assay to discriminate sperm quality beyond conventional semen parameters. A more detailed analysis revealed clear inter-individual differences between LBC and HBC donors, which were further supported by the hamster test. These findings suggest distinct levels of fertilization competence that may arise from intrinsic molecular features or subclinical pathologies. Interestingly, no significant correlation was found between total sperm motility prior to insemination and JUNO-binding efficiency during co-incubation. This finding contrasts with studies that associate motility with fertilization outcomes (Zinaman *et al.*, 2000; World Health Organization, 2021; Villani *et al.*, 2022). It is worth noting, however, that all semen samples analysed here were obtained from healthy donors and fulfilled the WHO reference values, thus representing a relatively homogeneous population. Within this restricted range, motility appears to lose its predictive value for fertilization potential. These observations support the notion that, although motility is important, it is not sufficient on its own to predict fertilization competence, reinforcing the need for functional assays that capture additional sperm attributes (Guzick *et al.*, 2001; Liu *et al.*, 2004; Mariappen *et al.*, 2018).

Collectively, these findings demonstrate that vitrification better preserves the structural and molecular integrity of sperm, enabling specific and functional interactions with JUNO-coated beads, while slow-freezing cryopreservation induces membrane and DNA damage that favours non-specific binding. Our bead-based model therefore emerges as a sensitive tool to discriminate between preservation methods and to identify spermatozoa with true fertilization competence. It also underscores the value of the JUNO-bead binding assay as a functional biomarker that goes beyond conventional semen analysis, providing a more precise assessment of the molecular competence of sperm to achieve fertilization.

## Supplementary Material

hoag010_Supplementary_Data

## Data Availability

All data needed to evaluate the conclusions in the paper are present in the paper and available in the institutional repository DIGITUM (University of Murcia) at http://hdl.handle.net/10201/181929.
