# Supplementary material for: JUNO-coated beads as a functional assay to capture and characterize fertilization-competent human sperm
Source: Hum Reprod Open. 2026 Feb 13;2026(1):hoag010. doi: 10.1093/hropen/hoag010 (PMC12961186; doi:10.1093/hropen/hoag010)
Supplement: hoag010_Supplementary_Data [file hoag010_supplementary_data.docx]

**Supplementary Table S1. Summary of the antibodies, commercial kits and software used in the study**

| **Reagents** | | **Stock concentration** | **Reference code** | **Source**  **(company or reference)** |
| --- | --- | --- | --- | --- |
| Antibodies | |  |  |  |
|  | Penta·His antibody, BSA free | 0.2 mg/mL | 34660 | Qiagen (Venlo, Netherlands) |
|  | Donkey anti-mouse IgG (H+L) HRP-conjugated | 0.04 mg/mL | SA1-100 | Thermo Fisher Scientific (Waltham, MA, USA) |
|  | Folate Receptor 4 (JUNO) Rabbit Polyclonal Antibody | 1 mg/mL | orb156918 | Biorbyt (Cambridge, United Kingdom) |
|  | Mouse anti-rabbit IgG-HRP | 0.4 mg/mL | sc-2357 | Santa Cruz Biotechnology (Dallas, TX, USA) |
|  | Donkey anti-mouse IgG (H+L) Alexa Fluor® 488-conjugated | 1.5 mg/mL | 715-546-150 | Jackson ImmunoResearch (West Grove, PA, USA) |
|  | Monoclonal mouse anti-IZUMO1 antibody (6F02) | 1 mg/mL | 6F02 | Tang et al., 2022 |
|  | Monoclonal mouse anti-IZUMO1 antibody (4E04) | 1 mg/mL | 4E04 | Tang et al., 2022 |
|  | Donkey anti-rabbit IgG (H+L) Alexa Fluor™ Plus 555-conjugated | 2 mg/mL | A32794 | Invitrogen (Carlsbad, CA, USA) |
|  | Monoclonal mouse anti-CD46 (M177 clone) FITC-conjugated | 0.2 mg/mL | sc-52647 | Santa Cruz Biotechnology (Dallas, TX, USA) |
| Kits | |  |  |  |
|  | Pierce™ ECL Plus Western Blotting Substrate |  | 32132 | Thermo Fisher Scientific (Waltham, MA, USA) |
|  | In Situ Cell Death Detection Kit, TMR red |  | 12156792910 | Roche (Basel, Switzerland) |
| Software | |  |  |  |
|  | Nicosystem Alto (version 2.5.2) |  |  | Nicoya (Kitchener, ON, Canada) |
|  | IDEAS 6.2 |  |  | Cytek Biosciences (Fremont, CA, USA) |
|  | BD FACSDiva |  |  | BD Biosciences (Milpitas, CA, USA) |
|  | RStudio (R version 4.1.3) |  |  | Posit PBC (Boston, MA, USA) |
|  | Leica Application Suite X (LAS X, version 3.10.0) |  |  | Leica Microsystems (Wetzlar, Germany) |

**Supplementary Table S2. Semen parameters of fresh samples**

|  |  | **Semen donors** | | |
| --- | --- | --- | --- | --- |
|  |  | M3 | M4 | M10 |
| **Baseline semen parameters** (standardized assessment in the clinic) | |  |  |  |
|  | Donor age (years) | 24 | 36 | 31 |
|  | Volume (mL) | 3.0 | 2.5 | 2.0 |
|  | pH | 8.5 | 8.7 | 8.7 |
|  | Concentration (M spz/mL) | 46.1 | 56.0 | 12.3 |
|  | Total motility (%) | 72 | 59 | 41 |
|  | Progressive motility (%) | 62 | 54 | 38 |
|  | Normal morphology (%) | 12 | 9 | 14 |
| **Post-processing sperm parameters** (lab assessment) | |  |  |  |
|  | Sperm concentration after Swim-up (M spz/mL) | 26 | 48 | 5 |
|  | Total motility after Swim-up (%) | 72 | 85 | 35 |
|  | Sperm concentration before co-incubation (M spz/mL) | 28 | 29 | 6 |
|  | Total motility before co-incubation (%) | 72 | 66 | 46 |

**Supplementary Table S3. Key parameters of binding performance at different co-incubation times, including mean number of spermatozoa bound to the bead and the percentage of bead with at least one sperm bound.**

| **Co-incubation time** | **BControl** | **BJUNO** | ***p* value** |
| --- | --- | --- | --- |
|  | Mean ± SE (n) | Mean ± SE (n) |  |
| 30 minutes | 0.02 ± 0.02 (91) | 0.5 ± 0.1 (94) | < 0.0001 |
| 1 hour | 0.03 ± 0.02 (93) | 1.3 ± 0.1 (98) | < 0.0001 |
| 2 hours | 0.3 ± 0.06 (93) | 1.5 ± 0.1 (97) | < 0.0001 |
| 4 hours | 0.6 ± 0.1 (91) | 1.8 ± 0.2 (95) | 0.0017 |
| Overnight | 0.5 ± 0.1 (91) | 4.8 ± 0.5 (90) | < 0.0001 |
|  | Percentage (CI 95%) | Percentage (CI 95%) |  |
| 30 minutes | 2.2 (0.4 – 8.5) | 36.2 (26.7 – 46.8) | < 0.0001 |
| 1 hour | 3.2 (0.8 – 9.8) | 65.3 (54.9 – 74.5) | < 0.0001 |
| 2 hours | 25.8 (17.5 – 36.1) | 74.2 (64.2 – 82.3) | < 0.0001 |
| 4 hours | 31.9 (22.7 – 42.6) | 73.7 (63.5 – 81.9) | 0.0028 |
| Overnight | 25.3 (17.0 – 35.7) | 91.1 (82.7 – 95.8) | < 0.0001 |

Abbreviations: BControl, beads control; BJUNO, beads JUNO; SE, standard error; CI, confidence interval.

**Supplementary Table S4. Donor characteristics and paired sperm parameters of the same semen samples preserved by cryopreservation and vitrification**

|  |  | **Semen donors** | | | | | |
| --- | --- | --- | --- | --- | --- | --- | --- |
|  |  | D045 | | D089 | | D048 | |
| **Biological Donor Parameters** | |  |  |  |  |  |  |
|  | Age (years) | 42 | | 19 | | 22 | |
|  | Blood group (ABO and Rh) | O- | | A+ | | A+ | |
|  | Proven fertility | Yes | | Yes | | Yes | |
| **Freezing method** | | Cryo | Vitri | Cryo | Vitri | Cryo | Vitri |
| **Post-thawing semen characteristics according to biobank** | |  |  |  |  |  |  |
|  | Progressive motility (%) | 40 | 78 | 42 | 86 | 33 | 77 |
|  | Normal morphology (%) | 9 | 9 | 6 | 6 | 8 | 8 |
| **Post-thawing semen characteristics (laboratory-assessed)** | |  |  |  |  |  |  |
|  | Sperm concentration after thawing (M spz/mL) | 34 | 7 | 15 | 5 | 12 | 12 |
|  | Total motility after thawing (%) | 14 | 44 | 29 | 48 | 12 | 57 |
|  | Sperm concentration after Swim-Up (M spz/mL) | 9 | 3 | 4 | 2 | 5 | 5 |
|  | Total motility after Swim-Up (%) | 64 | 64 | 54 | 65 | 53 | 81 |
|  | Sperm concentration before co-incubation (M spz/mL) | 4 | 2 | 2 | 3 | 4 | 4 |
|  | Total motility before co-incubation (%) | 60 | 52 | 63 | 75 | 58 | 62 |

Abbreviations: Cryo, Cryopreservation; Vitri, Vitrification

**Supplementary Table S5. Donor information, biobank and laboratory thawed semen parameters, and sperm-bead binding assay outcomes.**

| **Donor information** | | | | **Biobank thawing parameters**^a^ | | **Laboratory thawing parameters**^b^ | | | | | | **Experimental procedure** | | | |
| --- | --- | --- | --- | --- | --- | --- | --- | --- | --- | --- | --- | --- | --- | --- | --- |
| **SS** | **DA** | **BG** | **PF** | **PM** (%) | **NM** (%) | **SC_AT** (M /mL) | **TM_AT** (%) | **SC_ASU** (M /mL) | **TM_ASU** (%) | **SC_BI** (M /mL) | **TM_BI** (%) | **Spz_BJ** | **Spz_BC** | **Per_BJ** (%) | **Per_BC** (%) |
| D048_310123_P11 | 21 | A+ | Yes | 80 | 8 | 11 | 46 | 6 | 69 | 6 | 65 | 3.61 | 0.23 | 90.9 | 16.1 |
| D003_240323_P02 | 27 | AB+ | Yes | 65 | 7 | 6 | 41 | 4 | 55 | 2 | 62 | 6.00 | 0.43 | 90.6 | 33.3 |
| D053_031022_P04 | 26 | O+ | Yes | 63 | 7 | 11 | 39 | 3 | 75 | 2 | 57 | 3.87 | 0.37 | 86.7 | 19.4 |
| D048_070223_P03 | 21 | A+ | Yes | 70 | 8 | 3 | 27 | 1 | 68 | 1 | 75 | 1.95 | 0.30 | 78.4 | 24.2 |
| D003_240323_P03 | 27 | AB+ | Yes | 65 | 7 | 7 | 50 | 1 | 56 | 3 | 75 | 12.37 | 0.26 | 96.7 | 19.4 |
| D053_031022_P02 | 26 | O+ | Yes | 63 | 7 | 7 | 43 | 2 | 88 | 2 | 78 | 7.23 | 0.45 | 87.9 | 24.3 |
| D005_011222_P02 | 28 | A+ | Yes | 65 | 12 | 5 | 33 | 1 | 60 | 1 | 72 | 3.97 | 0.19 | 82.9 | 19.4 |
| D005_011222_P04+P01 | 28 | A+ | Yes | 65 | 12 | 4.5 | 30 | 6 | 55 | 6 | 59 | 7.19 | 0.50 | 100.0 | 40.0 |
| D063_041023_P05+P04 | 22 | O+ | Yes | 67 | 8 | 4.5 | 28 | 2 | 50 | 5 | 44 | 5.55 | 0.51 | 100.0 | 35.0 |
| D048_190923_P05+P06 | 22 | A+ | Yes | 67 | 8 | 5 | 22 | 2 | 64 | 4 | 75 | 9.48 | 1.10 | 100.0 | 56.7 |
| D048_090124_P01 | 22 | A+ | Yes | 77 | 8 | 6 | 34 | 4 | 46 | 2 | 70 | 2.69 | 0.18 | 86.7 | 17.7 |
| D045_090124_P01 | 42 | O- | Yes | 78 | 9 | 7 | 44 | 3 | 64 | 2 | 52 | 17.58 | 0.90 | 100.0 | 60.0 |
| D089_040124_P01 | 19 | A+ | Yes | 86 | 6 | 5 | 48 | 2 | 65 | 3 | 75 | 11.66 | 0.48 | 100.0 | 41.9 |
| D048_090124_P02+P03 | 22 | A+ | Yes | 77 | 8 | 12 | 57 | 5 | 81 | 4 | 62 | 4.00 | 1.11 | 93.6 | 63.0 |
| D045_090124_P02 | 42 | O- | Yes | 78 | 9 | 5 | 38 | 6 | 61 | 6 | 60 | 3.91 | 0.31 | 95.2 | 52.7 |
| D045_090124_P03 | 42 | O- | Yes | 78 | 9 | 3 | 43 | 2 | 47 | 4 | 44 | 3.18 | 0.24 | 90.9 | 21.0 |
| D059_220923_P05 | 24 | O+ | Yes | 78 | 9 | 4 | 28 | 3 | 60 | 4 | 60 | 14.10 | 0.31 | 96.6 | 22.9 |
| D040_120923_P05 | 23 | A+ | Yes | 65 | 6 | 5 | 23 | 8 | 85 | 6 | 70 | 13.70 | 0.09 | 100.0 | 6.2 |
| D048_070223_P01 | 21 | A+ | Yes | 70 | 8 | 7 | 45 | 3 | 50 | 2 | 55 | 2.47 | 0.10 | 90.6 | 10.0 |
| D089_040124_P02 | 19 | A+ | Yes | 86 | 6 | 3 | 55 | 4 | 50 | 4 | 56 | 25.39 | 0.31 | 100.0 | 21.9 |
| D089_040124_P03 | 19 | A+ | Yes | 86 | 6 | 5 | 47 | 4 | 60 | 4 | 61 | 5.59 | 0.33 | 96.6 | 24.2 |
| D059_220923_P04 | 24 | O+ | Yes | 78 | 9 | 2 | 31 | 2 | 40 | 2 | 42 | 11.64 | 1.09 | 100.0 | 56.3 |
| D048_190923_P07 | 22 | A+ | Yes | 67 | 8 | 6 | 28 | 2 | 60 | 2 | 58 | 7.79 | 0.13 | 100.0 | 12.5 |
| D040_120923_P03 | 23 | A+ | Yes | 65 | 6 | 6 | 37 | 4 | 66 | 5 | 60 | 11.04 | 0.21 | 100.0 | 20.7 |
| D105_090224_P03 | 21 | A+ | Yes | 55 | 6 | 10 | 55 | 7 | 74 | 5 | 61 | 2.63 | 0.00 | 80.0 | 0.0 |
| D045_120424_P01 | 42 | O- | Yes | 70 | 9 | 8 | 33 | 4 | 61 | 5 | 60 | 7.73 | 0.24 | 100.0 | 20.6 |
| D092_020224_P01 | 27 | O+ | Yes | 73 | 7 | 6 | 35 | 4 | 60 | 4 | 58 | 3.39 | 0.12 | 91.2 | 12.1 |
| D089_211223_P01 | 19 | A+ | Yes | 72 | 6 | 6 | 65 | 3 | 70 | 4 | 69 | 5.63 | 0.42 | 100.0 | 39.4 |
| D003_141122_P01 | 27 | AB+ | Yes | 75 | 7 | 9 | 45 | 4 | 68 | 5 | 70 | 16.00 | 0.52 | 96.0 | 28.6 |
| D045_150224_P13+P05 | 42 | O- | Yes | 75 | 9 | 10 | 50 | 4 | 58 | 4 | 48 | 10.67 | 1.29 | 100.0 | 62.5 |
| D040_250923_P08+P09 | 23 | A+ | Yes | 75 | 6 | 4 | 44 | 3 | 76 | 4 | 68 | 2.63 | 1.13 | 96.7 | 58.3 |
| D003_141122_P02+P03 | 27 | AB+ | Yes | 75 | 7 | 10 | 60 | 3 | 64 | 4 | 79 | 7.00 | 1.10 | 92.3 | 33.3 |
| D096_220224_P03+P04 | 30 | A+ | Yes | 67 | 7 | 7 | 32 | 2 | 51 | 4 | 50 | 6.13 | 0.26 | 90.0 | 22.6 |
| D045_150224_P06 | 42 | O- | Yes | 75 | 9 | 6 | 48 | 3.6 | 68 | 4 | 66 | 16.64 | 1.87 | 100.0 | 58.1 |
| D089_010923_P07 | 19 | A+ | Yes | 80 | 6 | 27 | 43 | - | - | 13 | 52 | 8.73 | 0.08 | 100.0 | 8.3 |
| D108_270224_P01+P02 | 34 | A+ | Yes | 55 | 6 | 11 | 50 | - | - | 4 | 41 | 4.80 | 0.21 | 94.0 | 21.0 |
| D040_031023_P05 | 27 | AB+ | Yes | 72 | 6 | 10 | 50 | - | - | 13 | 63 | 5.06 | 1.29 | 100.0 | 61.3 |
| D003_080923_P02 | 23 | A+ | Yes | 67 | 7 | 5 | 59 | - | - | 5 | 46 | 19.60 | 1.42 | 100.0 | 69.2 |
| D075_70224_P05 | 31 | A+ | Yes | 72 | 7 | 3 | 23 | - | - | 8 | 26 | 3.03 | 2.32 | 88.6 | 24.4 |
| D015_281024_P07 | 34 | O+ | No | 80 | 9 | 4 | 26 | - | - | 4 | 37 | 8.90 | 1.41 | 96.6 | 40.6 |
| D003_080923_P03 | 29 | AB+ | Yes | 67 | 7 | 3 | 53 | - | - | 3 | 56 | 5.19 | 0.90 | 96.8 | 44.8 |
| D133_131124_P02 | 29 | O+ | No | 65 | 9 | 3 | 55 | - | - | 4 | 78 | 8.67 | 0.88 | 100.0 | 38.5 |
| D108_070524_P07 | 35 | A+ | Yes | 62 | 6 | 3 | 66 | - | - | 4 | 46 | 18.25 | 0.39 | 100.0 | 32.3 |
| D032_140224_P01 | 26 | A+ | Yes | 65 | 9 | 6 | 35 | - | - | 12 | 41 | 15.93 | 1.70 | 100.0 | 63.3 |
| D015_281024_P02 | 34 | O+ | No | 80 | 9 | 12 | 50 | - | - | 13 | 44 | 4.20 | 1.41 | 93.3 | 55.6 |
| D108_070524_P08 | 35 | A+ | Yes | 62 | 6 | 12 | 54 | - | - | 12 | 44 | 6.50 | 1.00 | 100.0 | 45.5 |
| D108_070524_P09 | 35 | A+ | Yes | 62 | 6 | 7 | 75 | - | - | 5 | 38 | 11.07 | 0.44 | 96.4 | 32.4 |
| D003_080923_P05 | 29 | AB+ | Yes | 67 | 7 | 8 | 57 | - | - | 5 | 45 | 7.87 | 0.44 | 100.0 | 25.0 |
| D133_131124_P03 | 29 | O+ | No | 65 | 9 | 19 | 49 | - | - | 18 | 33 | 7.67 | 1.79 | 90.0 | 48.5 |
| D032_140224_P03 | 26 | A+ | Yes | 65 | 9 | 12 | 57 | - | - | 9 | 29 | 9.55 | 0.09 | 100.0 | 8.8 |
| D015_281024_P08 | 34 | O+ | No | 80 | 9 | 16 | 48 | - | - | 8 | 30 | 3.23 | 0.19 | 90.3 | 18.8 |
| D108_160224_P02 | 35 | A+ | Yes | 77 | 6 | - | - | 3.93 | 54.4 | 3.86 | 51.84 | 10.73 | 0.41 | 96.7 | 28.1 |
| D108_160224_P04 | 35 | A+ | Yes | 77 | 6 | - | - | 2.45 | 80.08 | 3.25 | 64.85 | 21.86 | 0.48 | 100.0 | 37.0 |
| D108_160224_P05 | 35 | A+ | Yes | 77 | 6 | - | - | 3.47 | 50.65 | 3.54 | 40.93 | 25.79 | 2.94 | 100.0 | 73.5 |
| D048_190923_P04 | 21 | A+ | Yes | 67 | 8 | - | - | 2.06 | 49.17 | 1.18 | 48.51 | 2.45 | 0.61 | 78.8 | 53.6 |
| D048_190923_P09 | 21 | A+ | Yes | 67 | 8 | - | - | 2.17 | 50.81 | 2.31 | 49.64 | 4.90 | 0.42 | 90.0 | 25.8 |
| D048_190923_P05 | 21 | A+ | Yes | 67 | 8 | - | - | 1.34 | 60.33 | 1.12 | 45.71 | 3.58 | 0.31 | 87.9 | 17.2 |

SS, Semen sample (Donor ID_ejaculate_straw); DA, Donor Age (years); BG, Blood Group (ABO and Rh); PF, Proven fertility; PM, Progressive motility (%); NM, Normal morphology (%); SC_AT, Sperm concentration after thawing (M spz/mL); TM_AT, Total motility after thawing (%); SC_ASU, Sperm concentration after Swim-Up (M spz/mL); TM_ASU, Total motility after Swim-Up (%); SC_BI, Sperm concentration before co-incubation (M spz/mL); TM_BI, Total motility before co-incubation (%); Spz_BJ, Mean number of spz bound to BJUNO; Spz_BC, Mean number of spz bound to Bcontrol; Per_BJ, BJUNO with ≥ 1 spz bound (%); Per_BC, Bcontrol with ≥ 1 spz bound (%); -, no data obtained.

^a^Semen parameters provided by the biobank company, assessed using a thawing test straw.

^b^Laboratory-assessed semen parameters after thawing the experimental straws.

| **A** | **B** |
| --- | --- |
| 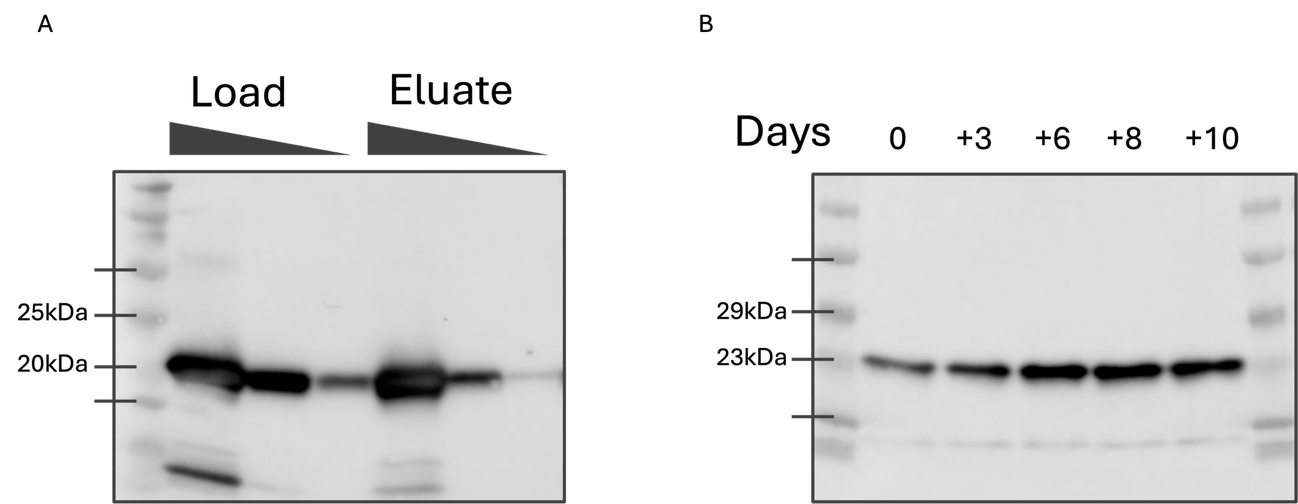 | 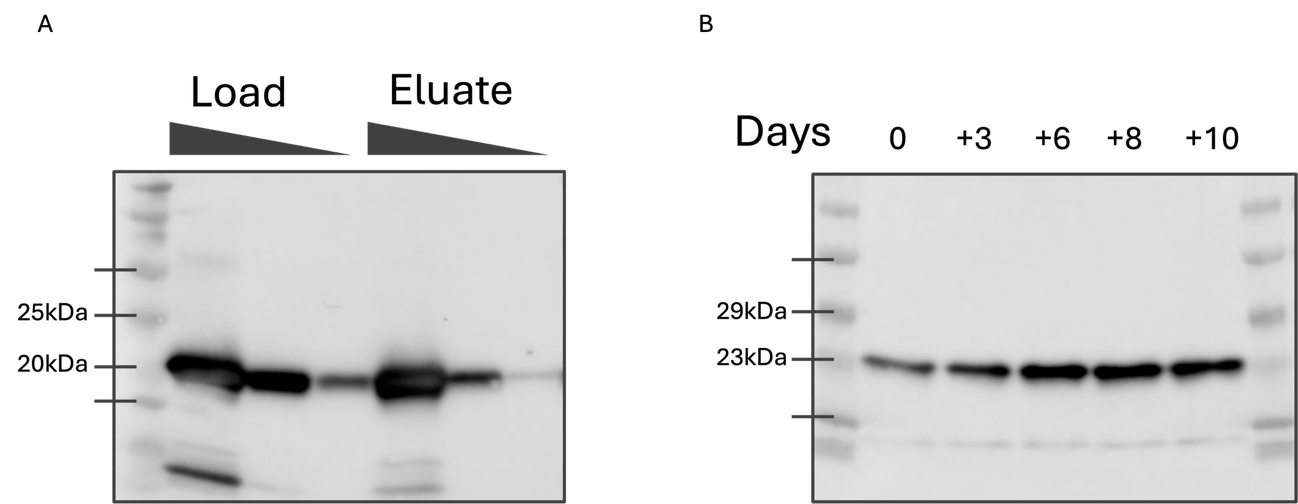 |

**Supplementary Figure S1**. **Recombinant human JUNO (rhJUNO) protein stable conjugated to beads**. **A**. Different rhJUNO protein concentrations for beads conjugation were evaluated by Western Blot. *Load* lanes indicate the decreasing amounts of rhJUNO protein tested for conjugation (3, 0.3 and 0.03 µg, respectively; ≈ 20 kDa). *Eluate* lanes represent the eluted fraction from 60 beads conjugated with each decreasing amounts of rhJUNO protein, confirming the graded reduction in protein content in the beads. **B**. The stability of protein conjugation on the beads during storage for up to 10 days at 4 °C in sodium phosphate buffer (the longest storage period employed) was evaluated by analyzing 60 beads on five alternate days after conjugation (days 0, 3, 6, 8, and 10).

| **A** | **B** |
| --- | --- |
| **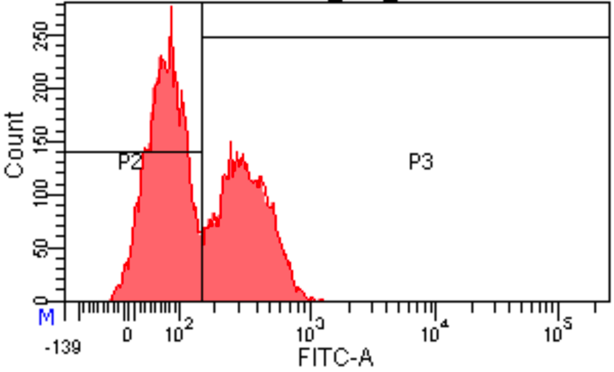** | **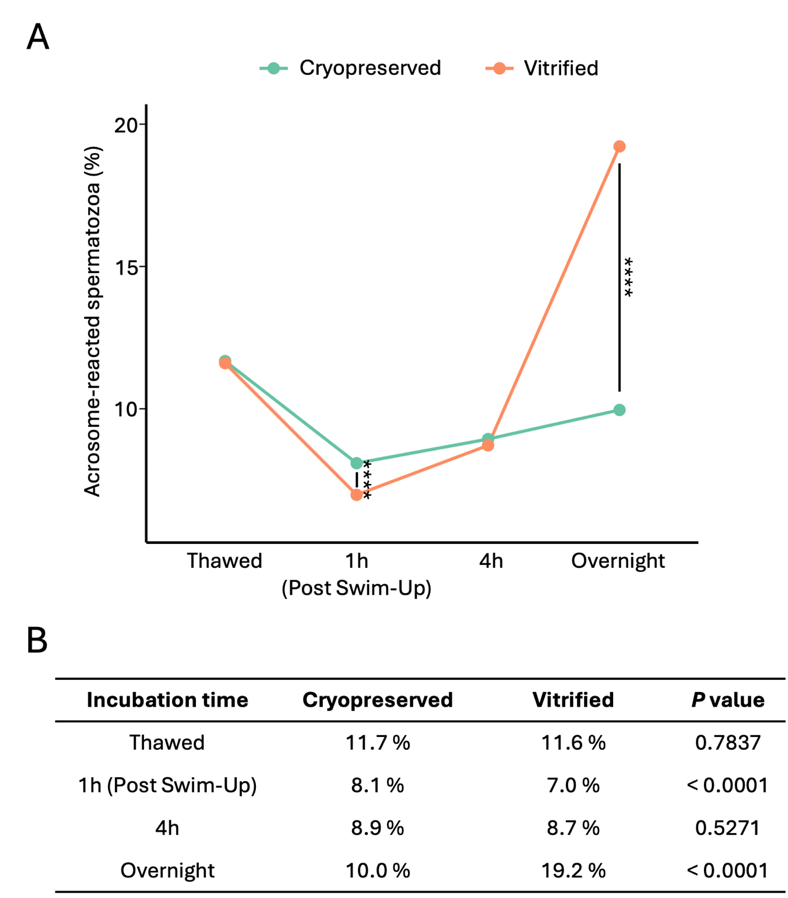** |
| **C**   \| **Incubation time** \| **Cryopreserved** (%) \| **Vitrified** (%) \| ***p* value** \| \| --- \| --- \| --- \| --- \| \| Thawed \| 11.7 \| 11.6 \| 0.7837 \| \| 1h (Post Swim-Up) \| 8.1 \| 7.0 \| <0.0001 \| \| 4h \| 8.9 \| 8.7 \| 0.5271 \| \| Overnight \| 10.0 \| 19.2 \| <0.0001 \| | |

**Supplementary Figure S2**. **Freezing method influences sperm acrosome reaction capacity**. **A**. Representative flow cytometry plot showing the gating strategy used to distinguish CD46-negative (P2) from CD46-positive (P3) cell populations, with the fluorescent signal arising from FITC-conjugated anti-CD46. **B.** Percentage of acrosome-reacted sperm cells over time under capacitating conditions at different time point, including immediately after thawing, sperm selection for 1 hour (Swim-Up), 4 hours and overnight incubation. *****p* ≤ 0.0001. **C**. Numerical values corresponding to panel B. Flow cytometry analysis reveals that vitrification better preserves the acrosome reaction capacity after overnight incubation.

**
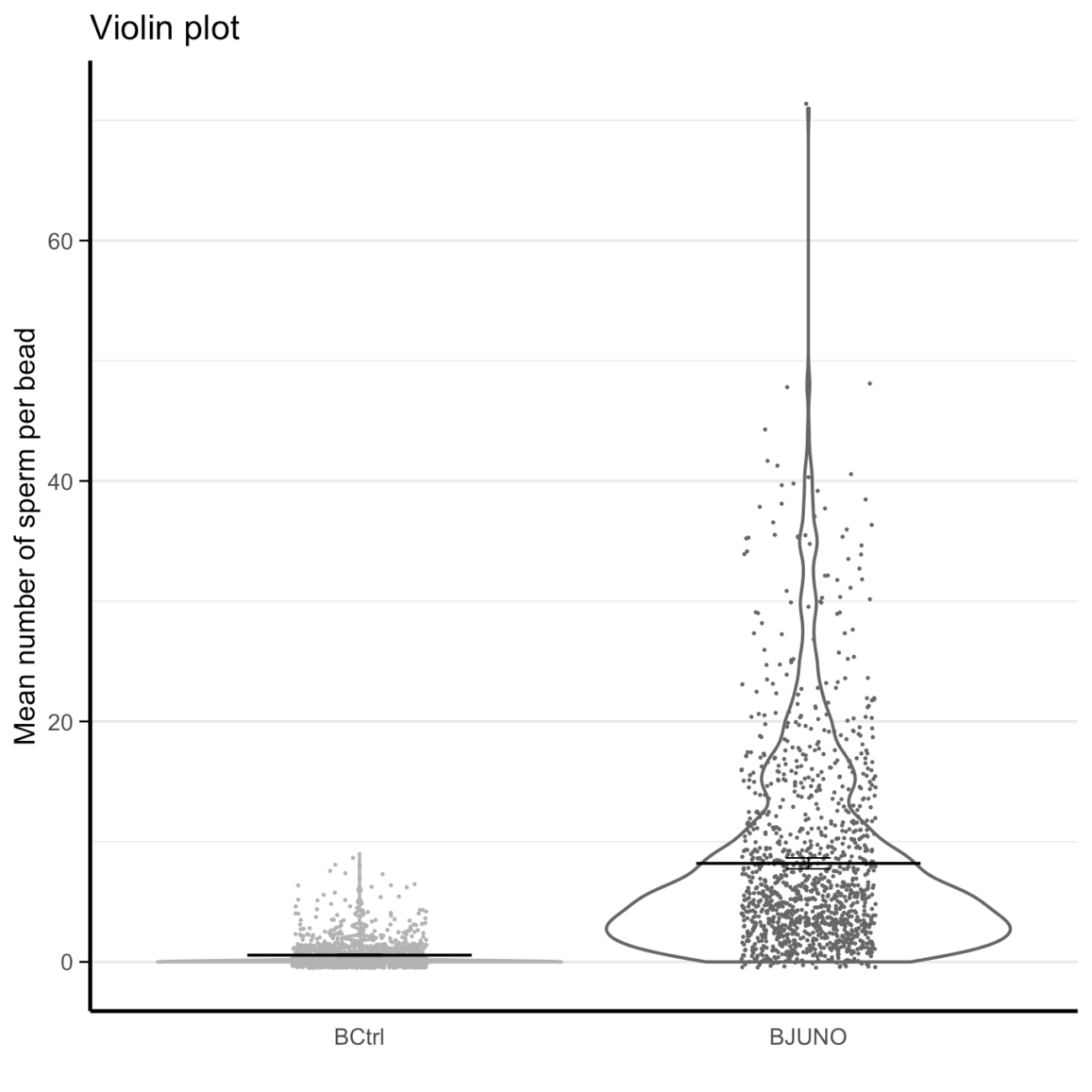
 Supplementary Figure S3**. **Human spermatozoa bind specifically to JUNO-coated beads.**Vitrified sperm from 18 donors (29 ejaculates) were tested, analyzing more than 3,300 beads. Each dot represents the number of spermatozoa bound to an individual bead. The mean number of spermatozoa bound to JUNO-coated beads was significantly higher than that bound to BControl.

| **A** | **B** |
| --- | --- |
| 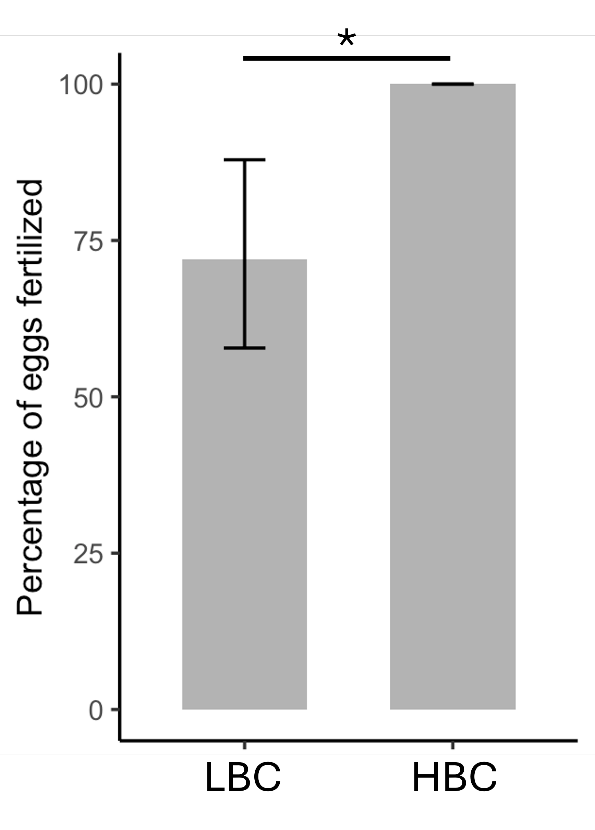 | 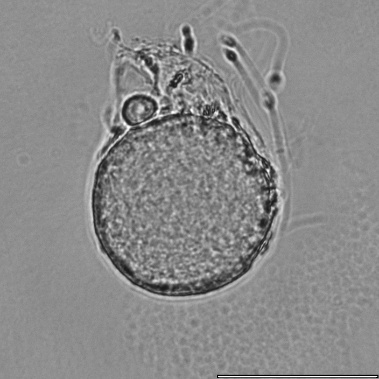 |
|  | 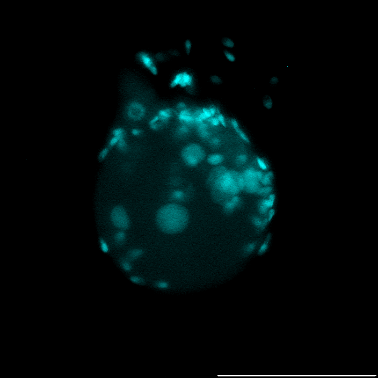 |

**Supplementary Figure S4. Hamster egg penetration test**. **A**. Percentage of eggs fertilized, as evidenced by sperm head decondensation, using sperm from donors classified as LBC (72.0%, n = 36) and HBC (100%, n = 35; p < 0.05). **B**. Representative images of a fertilized egg from an HBC donor: the upper panel shows the bright-field image and the lower panel shows the Hoechst-stained image, revealing several decondensed human sperm heads.
